# New Dimeric Members of the Phomoxanthone Family: Phomolactonexanthones A, B and Deacetylphomoxanthone C Isolated from the Fungus *Phomopsis* sp.

**DOI:** 10.3390/md11124961

**Published:** 2013-12-11

**Authors:** Bo Ding, Jie Yuan, Xishan Huang, Weitao Wen, Xu Zhu, Yayue Liu, Hanxiang Li, Yongjun Lu, Lei He, Hongmei Tan, Zhigang She

**Affiliations:** 1 School of Chemistry and Chemical Engineering, Sun Yat-sen University, 135 Xingang West Road, Guangzhou 510275, China; E-Mails: dingbo@mail2.sysu.edu.cn (B.D.); huangxishan13@foxmail.com (X.H.); liuyayue@mail2.sysu.edu.cn (Y.L.); lihanx@qq.com (H.L.); 2 Zhongshan School of Medicine, Sun Yat-sen University, 74 Zhongshan Road II, Guangzhou 510080, China; E-Mails: yuanjie@mail.sysu.edu.cn (J.Y.); wenwt@mail2.sysu.edu.cn (W.W.); zhuxun8@mail.sysu.edu.cn (X.Z.); 3 Key Laboratory of Tropical Disease Control (Sun Yat-sen University), Ministry of Education and Guangdong Province Key Laboratory of Functional Molecules in Oceanic Microorganism (Sun Yat-sen University), Bureau of Education, 74 Zhongshan Road II, Guangzhou 510080, China; 4 School of Life Sciences and Biomedical Center, Sun Yat-sen University, 135 Xingang West Road, Guangzhou 510275, China; E-Mails: luyj@mail.sysu.edu.cn (Y.L.); helei8688@126.com (H.L.)

**Keywords:** Mangrove, *Phomopsis* sp., Phomoxanthones, phomolactonexanthones, decetylphomoxanthone C, MTT assay

## Abstract

Three new phomoxanthone compounds, phomolactonexanthones A (**1**), B (**2**) and deacetylphomoxanthone C (**3**), along with five known phomoxanthones, including dicerandrol A (**4**), dicerandrol B (**5**), dicerandrol (**6**), deacetylphomoxanthone B (**7**) and penexanthone A (**8**), were isolated in the metabolites of the fungus *Phomopsis* sp. HNY29-2B, which was isolated from the mangrove plants. The structures of compounds **1**–**3** were established on the basis of spectroscopic analysis. All compounds were evaluated against four human cancer cell lines including human breast MDA-MB-435, human colon HCT-116, human lung Calu-3 and human liver Huh7 by MTT assay. The compounds **4**, ** 5**, **7** and **8** showed cyctotoxic activities against tested cancer cell lines (IC_50_ < 10 μM).

## 1. Introduction

Xanthones are simple tricyclic scaffold compounds that are mainly found as secondary metabolites in higher plants and microorganisms [1,2]. Xanthones have very diverse biological profiles, including antihypertensive, antioxidative, antithrombotic and anticancer activity, depending on the nature of their diverse structures and/or position of the different substitutes [3,4,5]. The xanthones have six different monoxanthones, including xanthone, two dihydroxanthones, two tetrahydroxanthones and hexahydroxanthone, which form various dimers of xanthone, dihydroxanthone and tetrahydroxanthone [5]. Phomoxanthone analogues, a kind of tetrahydroxanthone dimers, are argued the most structurally and biologically interesting xanthones from fungi [5,6].

The structure of phomoxanthone analogues are closely resembled the secalonic acids. However, a clear difference between phomoxanthone analogues and secalonic acids is that the carboxymethyl substituents at C-10a (C-10a′) have been replaced with the hydroxymethyl or acetoxymethyl substituents, and the C-5 (C-5′) hydroxyl moieties are acetylated [5,7]. Eight phomoxanthone analogues were isolated from fungus over the past 10 years. Phomoxanthones A and B were isolated from the fungus *phomosis* sp. BCC 1323, a teak endophyte collected from northern Thailand in 2001. They exhibited significant *in vitro* antimalarial and antitubercular activities and cytotoxicity [8]. Dicerandrols A, B and C were obtained from the fungus *phomosis longicolla* from Floridian rare mint species Dicerandra frutescens in 2001. These species had antimicrobial activities against *Bacillus sbutilis* and *Staphylococcus aureus* [9]. Deacetylphomoxanthone B was reported in 2008 as a metabolite from the *Phomosis* sp. PSU-D15, isolated from leave of Garcinia dulcis (Roxb.) Kurz in Songkhla Province, Thailand [10]. Monodeacytylphomoxanthone B was reported in 2013 as a new phomoxanthone antibiotic from *Phomosis Longicolla* S1B4 isolated from a plant sample in Hadong-gun Kyungam Provice, South Karea [11]. Penexanthone A was isolated from a *Penicillium* sp.CR1642D, collected from a Costa Rican rainforests in 2012 [12].

**Figure 1:**
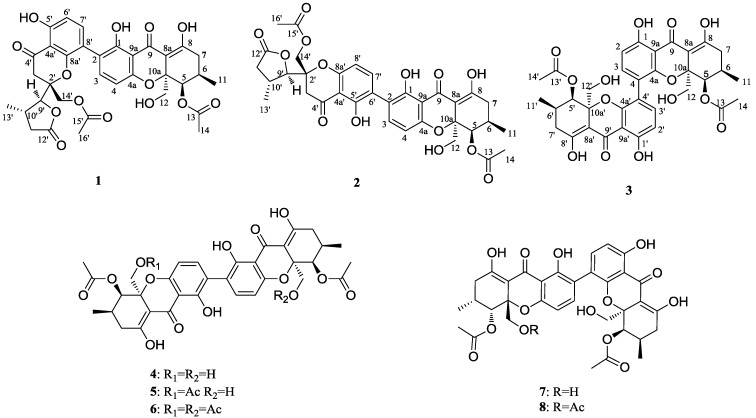
Compounds isolated from *Phomopsis* sp. HNY 29-2B (**1**–**8**).

During our ongoing search for bioactive constituents from marine fungi [13,14,15], we investigated the endophytic fungus *Phomosis* sp. HNY29-2B, isolated from a branch of mangrove *Acanthus ilicifolius* from the South China Sea. The fungus strain was cultured by solid substrate fermentation on rice. The fermentation mixture was extracted by methanol to afford a crude extract. Fractionation of the extract led to the isolation and structural determination of the three new phomoxanthones (**1**–**3**), which we named phomolactonexanthones A, B and deacetylphomoxanthone C, together with five known phomoxanthones (**4**–**8**) (Figure 1). Here, we report on the isolation, structural elucidation and antitumor activities of these phomoxanthones.

## 2. Results and Discussion

### 2.1. Structural Elucidation of New Compounds

**Figure 2:**
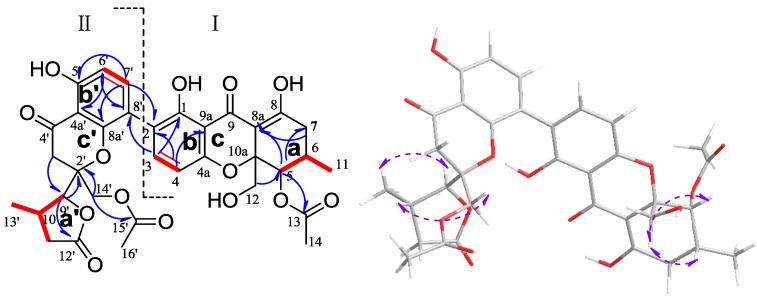
^1^H-^1^H COSY (red bold line), key HMBC (blue arrow) correlations and NOE (purple arrow) for compound **1**.

Phomolactonexanthone A (**1**) was an amorphous yellow powder with the molecular formula C_34_H_33_O_14_ by HRESIMS (calculated 665.18758 [M − H]^−^, found 665.18707). The IR spectra of **1** supported the presence of hydroxyl and carbonyl groups. A careful comparison of the ^1^H and ^13^C NMR data (Table 1) suggested that the compound **1** may be structurally related to the blennolide G [16]. It was also an asymmetric dimer of the usual tetrahydroxanthone I and an analogue of rearranged tetrahydroxanthone II (Figure 2). The monomer I of **1** in conjunction with the ^1^H-^1^H COSY, HSQC and HMBC data unambiguously verified three moieties: the first moiety was C-5/C-6(C-11)/C-7, the second moiety was C-12/C-10a/C-5 and the third moiety was C-3/C-4.The ring **a** of monomer I was indicated by the H-C long range correlations of H-7/C-8, C-8a; H-5/C-8a, C-10a, C-13; H-6/C-8; H-14/C-13, together with the first moiety and second moiety (Figure 2). Therefore, the carbons at δ_C_ 101.1 (C-8a) and δ_C_ 82.7 (C-10a) should be the position of attachment for the other ring. The H-C long range correlations of H-3/C-8′, C-4a, C-1; H-4/C-9a, C-2, C-4a, C-9 and 1-OH/C-9a, C-2, C-1, together with the third moiety, indicated the presence of ring **b** of monomer I (Figure 2). It was suggested that the carbons at δ_C_ 106.2 (C-9a) and δ_C_ 157.4 (C-4a) were the position of attachment for the other ring. Both of the H-4 and H-5 displayed the weak four-bond correlation to the carbonyl carbon at δ_C_ 187.7 (C-9), together with both of C-4a and C-10a shifting to the higher field, which verified the presence of ring **c** of monomer I (Figure 2). In the monomer II, the ^1^H-^1^H COSY and HMBC data unambiguously identified four moieties: the first moiety was C-9′/C-10′(C-13′)/C-11′/C-12′, the second moiety was C-6′/C-7′, the third moiety was C-4′/C-3′/C-2′/C-14′ and the fourth moiety was C-16′/C-15′. The H-C long range correlations of H-9′/C-12′, together with the first moiety, indicated the presence of γ-lactone ring **a′** of monomer II. The carbon at δ_C_ 87.3 (C-9′) was the position of attachment for the other ring (Figure 2). The H-C long range correlations of H-6′/C-4a′, C-8′, C-5′; H-7′/C-2, C-8a′, C-5′ and 5′-OH/C-4a′, C-6′, C-5′ suggested the presence of the ring **b′** of monomer II. It was indicated that the carbons at δ_C_ 107.2 (C-4a′) and δ_C_ 155.3 (C-8a′) were the position of attachment for the other ring. The H-C long range correlations of H-3′ α/C-9′; H-3′ β/ C-4a′; H-14′ α/ C-9′, C-15′ and H-9′/C-2′, together with the third and fourth moieties, indicated the presence of the ring **c′** of monomer II. The carbon at δ_C_ 87.3 (C-9′) was linked to the carbon at δ_C_ 82.2 (C-2′); the carbon at δ_C_ 155.3 (C-8a′) was linked with the carbon at δ_C_ 82.2 (C-2′) via the ether bond. The HMBC spectrum of H-C long range correlations of H-3/C-9a, C-1, C-8′ and H-7′/C-8a′, C-5′, C-2 indicated the presence of the C-2/C-8′ linkage in compound **1**. Finally, the monomer II was linked to the monomer I with the bond (C-2/C-8′). The molecular formula of compound **1** was identified, and compound **1** must be the 2, 8′ asymmetrical dimer (Figure 2). The stereochemistry of **1** was determined on the basis of 2D NOE analyses. A complete relative configuration of the molecule can not be given, as both the usual tetrahydroxanthone moiety and analogue of rearranged tetrahydroxanthone moiety of compound **1** were too far away in space. However, the independent relative configurations for each moiety can be determined. The NOEs correlation of H-12β/H-5, H-6 indicated the spatial relationship of the usual tetrahydroxanthone moiety, which relative configuration was thus characterized as 5R*, 6R*, 10aR*; the NOEs correlation of H_3_-13′/H-9′, H-10′/H-14′β indicated the spatial relationship of the analogue of rearranged tetrahydroxanthone moiety, which relative configuration was thus characterized as 2′R*, 9′S*, 10′S* (Figure 2).

**Table 1:** ^1^H and ^13^C NMR data for compounds **1** and **2** (500 MHz, in chloroform-*d*).

Position	1		2	
	δ_C_, Type	δ_H_, Mult. (*J* in Hz)	δ_C_, Type	δ_H_, Mult. (*J* in Hz)
1	159.4, C		159.6, C	
2	118.6, C		117.7, C	
3	139.6, CH	7.24, d (8.4)	140.2, CH	7.41, d (8.5)
4	108.2, CH	6.51, d (8.4)	108.0, CH	6.51, d (8.5)
4a	157.4, C		157.3, C	
5	70.2, CH	5.57, br s	70.3, CH	5.75, br s
6	27.9, CH	2.41, m	27.8, CH	2.40, m
7	33.5, CH_2_	2.42-2.50 m	33.5, CH_2_	2.41–2.48, m
8	178.7, C		178.1, C	
8a	101.1, C		101.0, C	
9	187.7, C		187.8, C	
9a	106.2, C		106.5, C	
10a	82.7, C		82.6, C	
11	17.6, CH_3_	1.08, d (6.0)	17.7, CH_3_	1.07, d (5.8)
12α	65.7, CH_2_	3.97, d (12.9)	65.7, CH_2_	4.09, d (13.0)
12β		3.58, d (12.9)		3.54, d (13.0)
13	170.6, C		170.7, C	
14	21.4, CH_3_	2.11, s	21.0, CH_3_	2.10, s
1-OH		11.84, s		11.86, s
2′	82.2, C		81.8, C	
3′α	38.9, CH_2_	3.37, d (17.3)	38.8, CH_2_	3.34, d (17.4)
3′β		2.76, d (17.4)		2.81, d (17.4)
4′	196.6, C		196.1, C	
4a′	107.2, C		107.2, C	
5′	161.5, C		159.1, C	
6′	110.3, CH	6.61, d (8.6)	117.9, C	
7′	140.3, CH	7.31, d (8.6)	140.8, CH	7.45, d (8.5)
8′	116.0, C		107.1, CH	6.45,d (8.5)
8a′	155.3, C		157.9, C	
9′	87.3, CH	4.10, d (2.7)	86.5, CH	4.23, d (3.7)
10′	29.3, CH	2.62, m	29.5, CH	2.92, m
11′ α	35.9, CH_2_	2.18, dd ( 17.8, 9.2)	36.6, CH_2_	2.94, m
11′ β		1.85, dd (17.8, 9.2)		2.24, m
12′	176.0, C		175.5, C	
13′	21.0, CH_3_	1.12, d (7.1)	20.6, CH_3_	1.28, d (6.7)
14′α	63.2, CH_2_	4.45, d (12.2)	63.8 CH_2_	4.39, d (12.1)
14′β		4.32, d (12.3)		4.31, d (12.1)
15′	169.9, C		170.0, C	
16′	20.6, CH_3_	2.03, s	20.9, CH_3_	2.03, s
5′-OH		11.63, s		11.98, s

**Figure 3:**
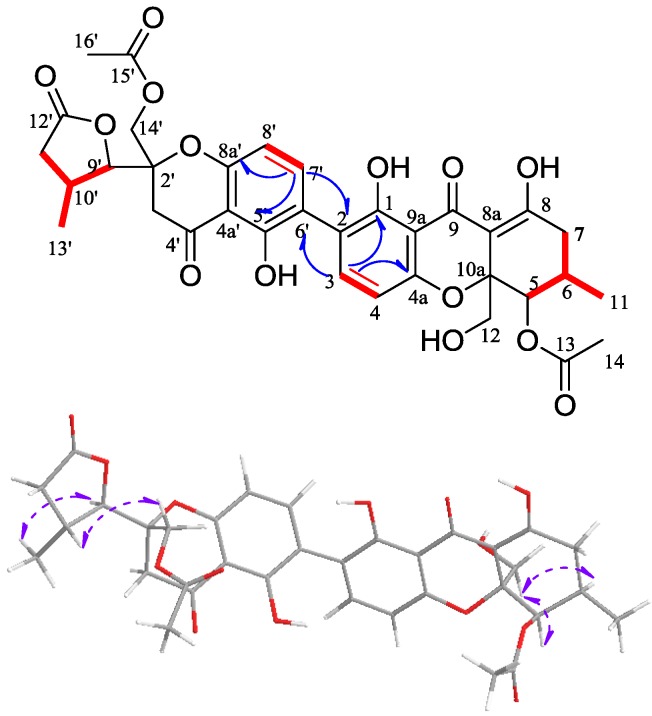
^1^H-^1^H COSY (red bold line), key HMBC (blue arrow) correlations and NOE (purple arrow) for compound **2**.

Phomolactonexanthone B (**2**) was also an amorphous yellow powder with the molecular formula C_34_H_33_O_14_ by HRESIMS (calculated 665.18758 [M − H]^−^, found 665.18731). The IR and UV spectra of phomolactonexanthone B were similar to those of phomolactonexanthone A. Analyses of 1D and 2D NMR spectra (Table 1 and Figure 3), particularly HMBC, revealed that this compound was also a dimer of the same monomers as in **1**, but the presence of different positions of attachment for dimerization. The HMBC spectrum of H-C long range correlations of H-3/C-4a, C-1, C-6′ and H-7′/C-8a′, C-5′, C-2 indicated the presence of the C-2/C-6′ linkage in compound **2**. The NMR assignments of protons and carbons of phomolactonexanthone B were established on basis of the connectivity network achieved by the analyses of ^1^H-^1^H COSY, HSQC and HMBC correlations. Also, the stereochemistry of **2** was determined on the basis of 2D NOE analyses (Figure 3). The independent relative configurations for the usual tetrahydroxanthone moiety and analogue of rearranged tetrahydroxanthone moiety can be determined as the compound **1**. The NOEs correlation of H-12β/H-6, H-5/H-12α, H-12β indicated the spatial relationship of the usual tetrahydroxanthone moiety, which relative configuration was thus characterized as 5R*, 6R*, 10aR*; the NOEs correlation of H_3_-13′/H-9′, H-10′/H-14′β, H-14′α indicated the spatial relationship of the analogue of rearranged tetrahydroxanthone moiety, which relative configuration was thus characterized as 2′S*, 9′R*, 10′R*.

**Figure 4:**
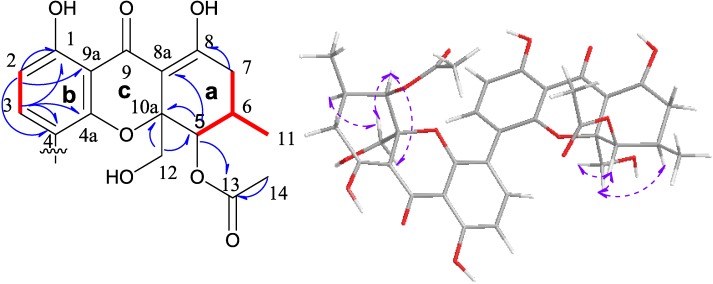
^1^H-^1^H COSY (red bold line) and key HMBC (blue arrow) correlations monomer of **3**, NOE (purple arrow) for compound **3**.

Deacetylphomoxanthone C (**3**) was an amorphous yellow powder with the molecular formula C_34_H_33_O_14_ by HRESIMS (calculated 665.18758 [M − H]^−^, found 665.18739). The presence of only 17 carbon signals in the ^13^C NMR spectrum (Table 2) indicated the symmetric, homodimer structure of this compound. The IR absorption at 3402 and 1614 cm^−1^ suggested the presence of hydroxyl and carbonyl groups. Analysis of the ^1^H and ^13^C NMR data for **3** indicated the presence of two carbonyls (an acetate at δ_C_ 169.9 and a ketone at δ_C_ 188.1), a tetrasubstituted aromatic ring, an enol, two methyls, two methylenes, two methines, and one quaternary carbon (δ_C_ 82.2). The presence of a moiety C-5/C-6(C-11)/C-7 [δ_C_ 17.6 (C-11), δ_C_ 69.7 (C-5), δ_C_ 27.9 (C-6), δ_C_ 33.3 (C-7); δ_H_ 1.01 (d, *J* = 6.4 Hz, H_3_-11), δ_H_ 5.58 (H-5), δ_H_ 2.27 (H-6), δ_H_ 2.37-2.41 (H_2_-7)], 5-acetoxyl [δ_C_ 21.0 (C-14), δ_C_ 169.9 (C-13), δ_C_ 69.7 (C-5); δ_H_ 2.14 (H_3_-14), δ_H_ 5.48 (H-5)], 10a-hydroxymethyl [δ_C_ 64.6 (C-12), δ_C_ 82.2 (C-10a); δ_H_ 3.87 (d, *J* = 13.0 Hz, H-12α), δ_H_ 3.42 (d, *J* = 13.0 Hz, H-12β)], a moiety C-2/C-3 [δ_C_ 110.2 (C-2), δ_C_ 141.1 (C-3); δ_H_ 6.58 (d, *J* = 8.6 Hz, H-2), δ_H_ 7.12 (d, *J* = 8.6 Hz, H-3)] moieties were unambiguously verified by the ^1^H-^1^H COSY and HMBC data (Figure 4). The H-C long range correlations of H_2_-7/C-8, C-8a; H-5/C-8a, C-10a, C-13, C-12; H-12α/C-5, C-10a; H-12β/C-10a; and 8-OH/ C-7, C-8a, C-8 indicated the presence of the ring **a**. The carbons at δ_C_ 101.5 (C-8a) and δ_C_ 82.2 (C-10a) were the position of attachment for the other ring. In addition, the H-C long range correlations of H-2/C-1, C-4, C-9a, C-9; H-3/ C-1, C-4a, C-4 and 1-OH/C-1, C2, C-9a suggested the presence of the ring **b**, which contained part of the enolized β-diketone system. Moreover, the carbon at δ_C_ 101.5 (C-8a) was linked to the carbon at δ_C_ 188.1 (C-9) and the carbon at δ_C_ 82.2 (C-10a) was linked with the carbon at δ_C_ 153.7 (C-4a) via the ether bond, which indicated the presence of the ring **c**. Then the monomer of **3** was verified (Figure 4), and the final substitution on the aromatic ring **b** was found to be the position of attachment for dimerization, and the only position available was C-4. Connecting with the HRESIMS data, compound **3** must be the 4,4′ symmetrical dimer (Figure 1). The relative configuration of **3** was determined using ^1^H-^1^H coupling constants and 2D NOESY experiment. According to the broad single peaks of H-5 and H-5′, protons H-5 and H-5′ should have a tinny coupling constant to H-6 and H-6′. It indicated that they had a *cis* disposition. In addition, the NOEs correlation of H-6/H-12β, H-5/H-12α, H-12β and H-6′/H-12′β, H-5′/H-12′α, H-12′β indicated their spatial relationship. This stereochemistry was analogous to the structure of the known phomoxanthone A [8]. Finally, the relative configuration of compound **3** was thus characterized as 5R*, 5′R*, 6R*, 6′R*, 10aR*, 10a′R* (Figure 4).

**Table 2:** ^1^H and ^13^C NMR data for compound **3** (400 MHz, in chloroform-*d*).

Position	δ_C_, Type	δ_H_, Mult. (*J* in Hz)
1,1′	161.8, C	
2,2′	110.2, CH	6.58, d (8.6)
3,3′	141.1, CH	7.12, d (8.6)
4,4′	115.4, C	
4a,4a′	153.7, C	
5,5′	69.7, CH	5.48, br s
6,6′	27.9, CH	2.27, m
7,7′	33.3, CH_2_	2.37-2.41, m
8,8′	177.7, C	
8a,8a′	101.5, C	
9,9′	188.1, C	
9a,9a′	107.1, C	
10a,10a′	82.2, C	
11,11′	17.6, CH_3_	1.01, d (6.4)
12α,12′α	64.6 CH_2_	3.87, d (13.0)
12β,12′β		3.42, d (13.0)
13,13′	169.9, C	
14,14′	21.0, CH_3_	2.14, s
1,1′-OH		11.44, s
8,8′-OH		13.97, s

The structures of **4**–**8** were identified by comparison of their spectroscopic data (UV, MS and NMR spectra) with those described in the literatures [9,10,12]. As the data agreed well with the values published (Supplementary Figures S13–S17), the structures of **4**–**8** were assigned as shown in Figure 1.

### 2.2. Cytotoxic Results

These phomoxanthone compounds (**1**–**8**) were evaluated for their cyctotoxic activity *in vitro* against four human cancer cell lines including human breast MDA-MB-435, human colon HCT-116, human lung Calu-3 and human liver Huh7, compared with their effects on the immortalized human breast epithelial cell line MCF-10A by MTT assay using epirubicin (an anticancer drug used widely in the clinic) as positive control [17]. The results are summarized in Table 3.

**Table 3:** Cytotoxicity IC_50_ (μM) of compounds **1**–**8** against MDA-MB-435, HCT-116, Calu-3, Huh7 and MCF-10A cell lines ^a^.

Coumpound	IC_50_ (μM)				
	MDA-MB-435 ^c^	HCT-116 ^c^	Calu-3 ^c^	Huh7 ^c^	MCF-10A ^c^
phomolactonexanthone A (**1**)	>50	>50	43.45 ± 2.51	>50	>50
phomolactonexanthone B (**2**)	>50	>50	>50	>50	>50
phomolactonexanthone C (**3**)	>50	44.06 ± 3.29	43.35 ± 2.09	>50	>50
dicerandrol A (**4**)	3.03 ± 0.12	2.64 ± 0.03	1.76 ± 0.02	4.19 ± 0.08	28.32 ± 3.57
dicerandrol B (**5**)	8.65 ± 0.66	3.94 ± 0.39	4.10 ± 0.08	30.37 ± 1.10	8.14 ± 1.27
dicerandrol C (**6**)	44.10 ± 2.45	42.63 ± 2.90	36.52 ± 3.32	>50	33.05 ± 2.74
deacetylphomoxanthone B (**7**)	14.40 ± 1.18	7.12 ± 0.70	4.14 ± 0.02	29.20 ± 1.19	>50
penexanthone A (**8**)	7.90 ± 0.58	6.92 ± 0.38	6.44 ± 0.86	42.82 ± 3.58	16.13 ± 1.57
epirubicin ^b^	0.56 ± 0.06	0.48 ± 0.029	1.03 ± 0.10	0.96 ± 0.01	0.48 ± 0.08

^a^ IC_50_ values are taken as means ± standard deviation from three independent experiment; ^b^ Used as a positive control. MDA-MB-435 ^c^, human breast cancer cell line; HCT-116 ^c^, human colon cancer cell line; Calu-3 ^c^, human lung cancer cell line; Huh7 ^c^, human liver cancer cell line; MCF-10A ^c^, human breast epithelial cell line.

These phomoxanthone analogues were tested on inhibitory activities against the growth of four tumor cell lines along with the immortalized human breast epithelial cell line. From the Table 3, the phomolactonexanthone A (**1**), phomolatonexanthone B (**2**) and deacetylphomoxanthone C (**3**) showed weak/no cytotoxic activities against tested cancer cell lines, but they displayed no cytotoxic effect on the immortalized human breast epithelial cell line. Dicerandrol A (**4**) showed broad-spectrum antitumor activity against MDA-MB-435, HCT-116, Calu-3 and Huh7 cell lines with IC50 values of 3.03, 2.64, 1.76 and 4.19 μM, respectively. However, Dicerandrol A also displayed cytotoxic effect on immortalized human breast epithelial cell line. The dicerandrol B (**5**) and penexanthone A (**8**) exhibited marked cytotoxic activities against MDA-MB-435, HCT-116 and Calu-3 (IC_50_ < 10 μM), and displayed less marked cytotoxic effect on the immortalized human breast epithelial cell line. Moreover Deacetylphomoxanthone B (**7**) displayed excellent selective activities against HCT-116 and Calu-3 cell lines over other cancer cell lines and showed no cytotoxic effect on the immortalized human breast epithelial cell line. In addition, Dicerandrol A (**4**) possessed the most potent activity against human live cell line Huh7, but it was reported that dicerandrol A exhibited less cytotoxic activities than dicerandrol B across the two cancer cell lines, HCT-116 and A549 (colon and lung tumor, respectively) [9]. Dicerandrols A and B displayed marked anticancer activities against MDA-MB-435, HCT-116 and Calu-3, while Dicerandrol C exhibited weak anticancer activities. The activity profiles suggested that free hydroxyl groups at C-12 and/or C-12′ of phomoxanthones was the key pharmacophore of Dicerandrols.

## 3. Experimental Section

### 3.1. General

Melting points were determined on a Fisher Johns hot-stage apparatus and are uncorrected. Optical rotations were measured on a MCP 300 (Anton Paar, Graz, Austria) polarimeter at 25 °C. IR spectra were measured on a Nicolet 5DX-FTIR spectrophotometer. UV spectra were measured on the HPLC (Waters 1525, Westbrook, CT, USA) with PDA detection (Waters 2898, Greenville, SC, USA). NMR data were recorded on Bruker Avance 400, 600 and 500 spectrometers using TMS as an internal standard. HRMS data were measured on LTQ Orbitrap LC-MS (Thermo, Rockford, IL, USA). Column chromatography was performed using silica gel (200–300 mesh, Qingdao Marine Chemcials, Qingdao, China). Semipreparative HPLC was operated on a Waters 1525 LC using an Ultimate XB-C18 column (250 × 10 mm, 5 μm; Welch, Shanghai, China).

### 3.2. Fungal Material

The fungus strain HNY29-2B was isolated from a branch of mangrove plant *Acanthus llicifolius* collected from the South China Sea in Hainan province, China. It was identified as *Phomopsis* sp. by Dr. Yayue Liu at School of Chemistry and Chemical Engineering Sun Yat-sen University, and the sequence data have submitted to GenBank (accession no. KF387574). A voucher specimen (registration number HNY29-2B) was deposited at Sun Yat-sen University, Guangdong, China.

### 3.3. Extraction and Isolation

The fungus was fermented onto auto autoclaved rice solid-substrate medium (thirty 1000 mL Erlenmeyer flasks, each containing 80 g of rice and 80 mL of distilled water) for 5 weeks at 25 °C. The fermentation mixture was broken up with a spatula and extracted three times with methanol. The methanol layer was filtered and evaporated to yield crude extract (50.9 g).

The crude extract was partitioned with petroleum ether (PE), dichloromethane and ethyl acetate (EA). The dichloromethane fraction (25.3 g) was separated by silica gel column chromatography using gradient elution with a mixture of PE and EA to obtain 10 fractions (D1–D12). D2 (6.7 g) was subsequently subjected to silica gel column chromatography eluting with PE-EA (7:1) to yield **5** (1.3 g). D4 (3.8 g) was treated according to the same procedure as D2 to yield **4** (62 mg), and **7** (46 mg). From D3 (620 mg) and D5 (310 mg) fractions, compound **1** (4.2 mg), compound **2** (6.0 mg), compound **3** (6.4 mg), compound **8** (13 mg) and compound **6** (10.7 mg) were separated upon semi-preparative HPLC using a gradient elute (MeOH–H_2_O).

Phomolactonexanthone A (**1**): Yellow powder, melting point: 102.1–103.2 °C, 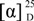 −75 (c, 0.2, MeOH), IR ν_max_ (KBr): 3428, 2917, 2521, 2138, 1798, 1635, 1436, 1388, 1061 cm^−1^, UV (PDA) λ_max_ 241.6, 340.3 nm; for ^1^H and ^13^C NMR data see Table 1; HRESIMS *m/z* 665.18707 [M − H]^−^ (calcd for C_34_H_33_O_14_, 665.18758).

Phomolactonexanthone B (**2**): Yellow powder, melting point: 101.6–102.4 °C, 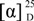 −21.7 (c, 0.6, MeOH), IR ν_max_ (KBr): 3429, 2919, 2516, 1796, 1628, 1434, 1057 cm^−1^, UV (PDA) λ_max_ 245.2, 340.3 nm; for ^1^H and ^13^C NMR data see Table 1; HRESIMS *m/z* 665.18731 [M − H]^−^ (calcd for C_34_H_33_O_14_, 665.18758).

Deacetylphomoxanthone C (**3**): Yellow powder, melting point: 145.5–146.7 °C, 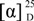 +101.6 (c, 0.44, MeOH), IR ν_max_ (KBr): 3402, 2925, 1743, 1614, 1465, 1366, 1224, 1045 cm^−1^, UV (PDA) λ_max_ 216.9, 248.7, 337.9 nm; for ^1^H and ^13^C NMR data see Table 2; HRESIMS *m/z* 665.18739 [M − H]^−^ (calcd for C_34_H_33_O_14_, 665.18758).

### 3.4. Antitumor Activity *in Vitro*

#### 3.4.1. Cell Culture

MDA-MB-435, HCT-116, Calu-3 and Huh7 were cultured in Dulbecco’s modification Eagle’s medium (DMEM, Invitrogen, Carlsbad, CA, USA) supplemented with 10% fetal bovine serum (FBS, Hyclone, Logan, UT, USA), 2 mM l-glutamine, 100 μg/mL streptomycin and 100 U/mL penicillin (Invitrogen, Carlsbad, CA, USA). MCF-10A cells were cultured in keratinocyte serum free medium (KSFM) supplemented with 0.1–0.2 ng/mL human recombinant epidermal growth factor and 20–30 μg/mL bovine pituitary extract (Invitrogen, Carlsbad, CA, USA). The cells were incubated at 37 °C in a humidified atmosphere with 5% CO_2_.

#### 3.4.2. Assessment of Antitumor Activity by MTT Assay

Cells were harvested during logarithmic growth phase and seeded in 96-well plates at a density of 1 × 104 cells/mL, and cultured at 37 °C in a humidified incubator (5% CO_2_) for 24 h, followed by exposure to various concentrations of compounds tested for 48 h. Subsequently 20 μL of MTT (Genview, Houston, TX, USA) solution (5 mg/mL) was added to each well and mixed, the cells were then incubated for an additional 4 h. Culture supernatant was removed using the micropipette, 150 μL of DMSO (Sangon Biotech, Shanghai, China) was added to each well to fully dissolve the MTT-formazan crystals. Cell growth inhibition was determined by measuring the absorbance (Abs) at λ = 570 nm using a microplate reader and calculated according to the following equation:

Growth inhibition = (1 − OD of treated cells/OD of control cells) × 100%

The half maximal inhibitory concentrations (IC_50_) were obtained from linear regression analysis of the concentration-response curves plotted for each tested compound.

## 4. Conclusions

The present work was isolation, structure determination and biological activity of three new phomoxanthone analogues and five known compounds. The phomolactonexanthones A and B are two new phomoxanthone analogues, thereby enlarging the phomoxanthone family. Dicerandrol A (**4**) showed significant cytotoxic activities against all the tested cancer cell lines. Dicerandrol B (**5**), Deacetylphomoxanthone B (**7**) and penexanthone A (**8**) have selectivity in the antitumor biological assay. The cytotoxic results revealed that some of the phomoxanthone analogues are a potential antitumor drug and/or lead to compounds for constructing an antitumor compound library.

## Supplementary Files

Supplementary File 1 Supplementary Information (PDF, 7459 KB)

## References

[1] Hostettmann K., Wagner H. (1977). Xanthone glycosides. Phytochemistry.

[2] Na Y. (2009). Recent cancer drug development with xanthone structures. J. Pharm. Pharmacol..

[3] Pinto M.M.M., Sousa M.E., Nascimento M.S.J. (2005). Xanthone Derivatives: New Insights in Biological Activities. Curr. Med. Chem..

[4] Yang C.-H., Ma L., Wei Z.-P., Han F., Gao J. (2012). Advances in Isolation and Synthesis of Xanthone Derivatives. Chin. Herb. Med..

[5] Masters K.-S., Bräse S. (2012). Xanthones from Fungi, Lichens, and Bacteria: The Natural Products and Their Synthesis. Chem. Rev..

[6] Lim C., Kim J., Choi J.N., Ponnusamy K., Jeon Y., Kim S.U., Kim J.G., Lee C. (2010). Identification, fermentation, and bioactivity against *Xanthomonas oryzae* of antimicrobial metabolites isolated from *Phomopsis longicolla* S1B4. J. Microbiol. Biotechnol..

[7] Elsässer B., Krohn K., Flörke U., Root N., Aust H.-J., Draeger S., Schulz B., Antus S., Kurtán T. (2005). X-ray Structure Determination, Absolute Configuration and Biological Activity of Phomoxanthone A. Eur. J. Org. Chem..

[8] Isaka M., Jaturapat A., Rukseree K., Danwisetkanjana K., Tanticharoen M., Thebtaranonth Y. (2001). Phomoxanthones A and B, Novel Xanthone Dimers from the Endophytic Fungus *Phomopsis* Species. J. Nat. Prod..

[9] Wagenaar M.M., Clardy J. (2001). Dicerandrols, New Antibiotic and Cytotoxic Dimers Produced by the Fungus *Phomopsis longicolla* Isolated from an Endangered Mint. J. Nat. Prod..

[10] Rukachaisirikul V., Sommart U., Phongpaichit S., Sakayaroj J., Kirtikara K. (2008). Metabolites from the endophytic fungus *Phomopsis* sp. PSU-D15. Phytochemistry.

[11] Choi J.N., Kim J., Ponnusamy K., Lim C., Kim J.G., Muthaiya M.J., Lee C.H. (2013). Identification of a new phomoxanthone antibiotic from *Phomopsis longicolla* and its antimicrobial correlation with other metabolites during fermentation. J. Antibiot..

[12] Cao S., McMillin D.W., Tamayo G., Delmore J., Mitsiades C.S., Clardy J. (2012). Inhibition of Tumor Cells Interacting with Stromal Cells by Xanthones Isolated from a Costa Rican *Penicillium* sp.. J. Nat. Prod..

[13] Wen L., Cai X., Xu F., She Z., Chan W.L., Vrijmoed L.L.P., Jones E.B.G., Lin Y. (2009). Three Metabolites from the Mangrove Endophytic Fungus *Sporothrix* sp. (#4335) from the South China Sea. J. Org. Chem..

[14] Huang X., Huang H., Li H., Sun X., Huang H., Lu Y., Lin Y., Long Y., She Z. (2013). Asperterpenoid A, a New Sesterterpenoid as an Inhibitor of Mycobacterium tuberculosis Protein Tyrosine Phosphatase B from the Culture of *Aspergillus* sp. 16-5c. Org. Lett..

[15] Xiao Z.E., Huang H.R., Shao C.L., Xia X.K., Ma L., Huang X.S., Lu Y.J., Lin Y.C., Long Y.H., She Z.G. (2013). Asperterpenols A and B, New Sesterterpenoids Isolated from a Mangrove Endophytic Fungus *Aspergillus* sp 085242. Org. Lett..

[16] Zhang W., Krohn K., Zia U., Flörke U., Pescitelli G., Di Bari L., Antus S., Kurtán T., Rheinheimer J., Draeger S. (2008). New Mono- and Dimeric Members of the Secalonic Acid Family: Blennolides A–G Isolated from the Fungus *Blennoria* sp.. Chem. Eur. J..

[17] Chen H., Zhong L., Long Y., Li J., Wu J., Liu L., Chen S., Lin Y., Li M., Zhu X. (2012). Studies on the Synthesis of Derivatives of Marine-Derived Bostrycin and Their Structure-Activity Relationship against Tumor Cells. Mar. Drugs.

