# Supplementary material for: New Dimeric Members of the Phomoxanthone Family: Phomolactonexanthones A, B and Deacetylphomoxanthone C Isolated from the Fungus Phomopsis sp
Source: Mar Drugs. 2013 Dec 11;11(12):4961–72. doi: 10.3390/md11124961 (PMC3877896; doi:10.3390/md11124961)

Supplementary Information

Table S1. NMR data for compounds **1** (500 MHz, in chloroform-*d*).
Table S2. NMR data for compounds **2**.
Table S3. NMR data for compounds **3**.
Figure S1. ^{1}H, ^{13}C, Dept 135, ^{1}H-^{1}H COSY, HSQC, HMBC, NOE of compound **1**.
Figure S2. HRESIMS of compound **1**.
Figure S3. IR of compound **1**.
Figure S4. UV of compound **1**.
Figure S5. ^{1}H, ^{13}C, ^{1}H-^{1}H COSY, HSQC, HMBC, NOE of compound **2**.
Figure S6. HRESIMS of compound **2**.
Figure S7. IR of compound **2**.
Figure S8. UV of compound **2**.
Figure S9. ^{1}H, ^{13}C, Dept 135, ^{1}H-^{1}H COSY, HSQC, HMBC, NOE of compound **3**.
Figure S10. HRESIMS of compound **3**.
Figure S11. IR of compound **3**.
Figure S12. UV of compound **3**.
Figure S13. ^{1}H, ^{13}C, ^{1}H-^{1}H COSY, HSQC, HMBC, NOE of compound **4**.
Figure S14. ^{1}H, ^{13}C, ^{1}H-^{1}H COSY, HSQC, HMBC, NOE of compound **5**.
Figure S15. ^{1}H, ^{13}C, ^{1}H-^{1}H COSY, HSQC, HMBC, NOE of compound **6**.
Figure S16. ^{1}H, ^{13}C, ^{1}H-^{1}H COSY, HSQC, HMBC, NOE of compound **7**.
Figure S17. ^{1}H, ^{13}C, ^{1}H-^{1}H COSY, HSQC, HMBC, NOE of compound **8**.

Table S1. NMR data for compounds **1** (500 MHz, in chloroform-*d*).

Position	δ_C, Type	δ_H, Mult. (*J* in Hz)	HMBC(H→C)	COSY
1	159.4, C			
2	118.6, C			
3	139.6, CH	7.24, d (8.4)	8′,1,4a	H-4
4	108.2, CH	6.51, d (8.4)	9a,2,4a,9	H-3
4a	157.4, C			
5	70.2, CH	5.57, br s	11,6,7,8a,10a,13,9	H-6
6	27.9, CH	2.41, m	11,8a,8	H-5, H-11
7	33.5, CH_2	2.42–2.50 m	6,5,8a,8	
8	178.7, C			
8a	101.1, C			
9	187.7, C			
9a	106.2, C			
10a	82.7, C			
11	17.6, CH_3	1.08, d (6.0)	6,7,5	H-6
12α	65.7, CH_2	3.97, d (12.9)	5,10a	
12β		3.58, d (12.9)		
13	170.6, C			
14	21.4, CH_3	2.11, s	13	
1-OH		11.84, s	9a,2,1	
2′	82.2, C			
3′α	38.9, CH_2	3.37, d (17.3)	2′,9′,14′,4′	
3′β		2.76, d (17.4)	4a′4′	
4′	196.6, C			
4a′	107.2, C			
5′	161.5, C			
6′	110.3, CH	6.61, d (8.6)	4a′,8′,5′	
7′	140.3, CH	7.31, d (8.6)	2,8a′,5′	
8′	116.0, C			
8a′	155.3, C			
9′	87.3, CH	4.10, d (2.7)	10′,13′,12′,2′	H-10′
10′	29.3, CH	2.62, m		H-11′,H-9′,H-13′
11′α	35.9, CH_2	2.18, dd (17.8, 9.2)	12′,10′	H-10′
11′β		1.85, dd (17.8, 9.2)		
12′	176.0, C			
13′	21.0, CH_3	1.12, d (7.1)	10′,11′,9′	H-10′
14′α	63.2, CH_2	4.45, d (12.2)	3′2′,9′,15′	
14′β		4.32, d (12.3)		
15′	169.9, C			
16′	20.6, CH_3	2.03, s	15′	
5′-OH		11.63, s	6′,4a′,5′	

Table S2. NMR data for compounds **2**.

Position	δ_C, Type	δ_H, Mult. (*J* in Hz)	HMBC(H→C)	COSY
1	159.6, C			
2	117.7, C			
3	140.2, CH	7.41, d (8.5)	6′,4a,1	H-4
4	108.0, CH	6.51, d (8.5)	9a,2,4a,9	H-3
4a	157.3, C			
5	70.3, CH	5.75, br s	11,6,7,8a, 12,10a,13,9	H-6
6	27.8, CH	2.40, m	11,7	H-5,H-11
7	33.5, CH_2	2.41–2.48, m	6,5,8a,8	
8	178.1, C			
8a	101.0, C			
9	187.8, C			
9a	106.5, C			
10a	82.6, C			
11	17.7, CH_3	1.07, d (5.8)	6,7,5,8	H-6
12α	65.7, CH_2	4.09, d (13.0)	5,10a,8a	
12β		3.54, d (13.0)	5	
13	170.7, C			
14	21.0, CH_3	2.10, s	13	
1-OH		11.86, s	9a,2,1	
2′	81.8, C			
3′α	38.8, CH_2	3.34, d (17.4)	2′,9′,14′,4′	
3′β		2.81, d (17.4)	4a′4′14′	
4′	196.1, C			
4a′	107.2, C			
5′	159.1, C			
6′	117.9, C			
7′	140.8, CH	7.45, d (8.5)	2,8a′,5′	H-8′
8′	107.1, CH	6.45,d (8.5)	4a′,6′,8a′	H-7′
8a′	157.9, C			
9′	86.5, CH	4.23, d (3.7)	13′,12′	H-10′
10′	29.5, CH	2.92, m		H-11′,H-9′,H-13′
11′α	36.6, CH_2	2.94, m	12′,10′,13′	H-10′
11′β		2.24, m	13′,10′,9′,12′	
12′	175.5, C			
13′	20.6, CH_3	1.28, d (6.7)	10′,11′,9′	H-10′
14′α	63.8 CH_2	4.39, d (12.1)	3′2′,9′,15′	
14′β		4.31, d (12.1)		
15′	170.0, C			
16′	20.9, CH_3	2.03, s	15′	
5′-OH		11.98,s	6′,4a′,5′	

Table S3. NMR data for compounds **3**.

Position	δ_C, Type	δ_H, Mult. (*J* in Hz)	HMBC(H→C)	COSY
1,1′	161.8, C			
2,2′	110.2, CH	6.58, d (8.6)	1,4,9a,4a,9	H-3
3,3′	141.1, CH	7.12, d (8.6)	1,4a,4	H-2
4,4′	115.4, C			
4a,4a′	153.7, C			
5,5′	69.7, CH	5.48, br s	13,8a,10a, 7,11,6,12	H-6
6,6′	27.9, CH	2.27, m		H-5,H-11,H-7
7,7′	33.3, CH_2	2.37–2.41, m	8,8a,5,6,11	H-6
8,8′	177.7, C			
8a,8a′	101.5, C			
9,9′	188.1, C			
9a,9a′	107.1, C			
10a,10a′	82.2, C			
11,11′	17.6, CH_3	1.01, d (6.4)	6,7,5	H-6
12α,12′α	64.6 CH_2	3.87, d (13.0)	5,10a	
12β, 12′β		3.42, d (13.0)	10a	
13,13′	169.9, C			
14,14′	21.0, CH_3	2.14, s	13	
1,1′-OH		11.44, s	1,9a,2	
8,8′-OH		13.97, s	7,8a,8	

Figure S1. ^{1}H, ^{13}C, Dept 135, ^{1}H-^{1}H COSY, HSQC, HMBC, NOE of compound **1**.

^{1}H NMR

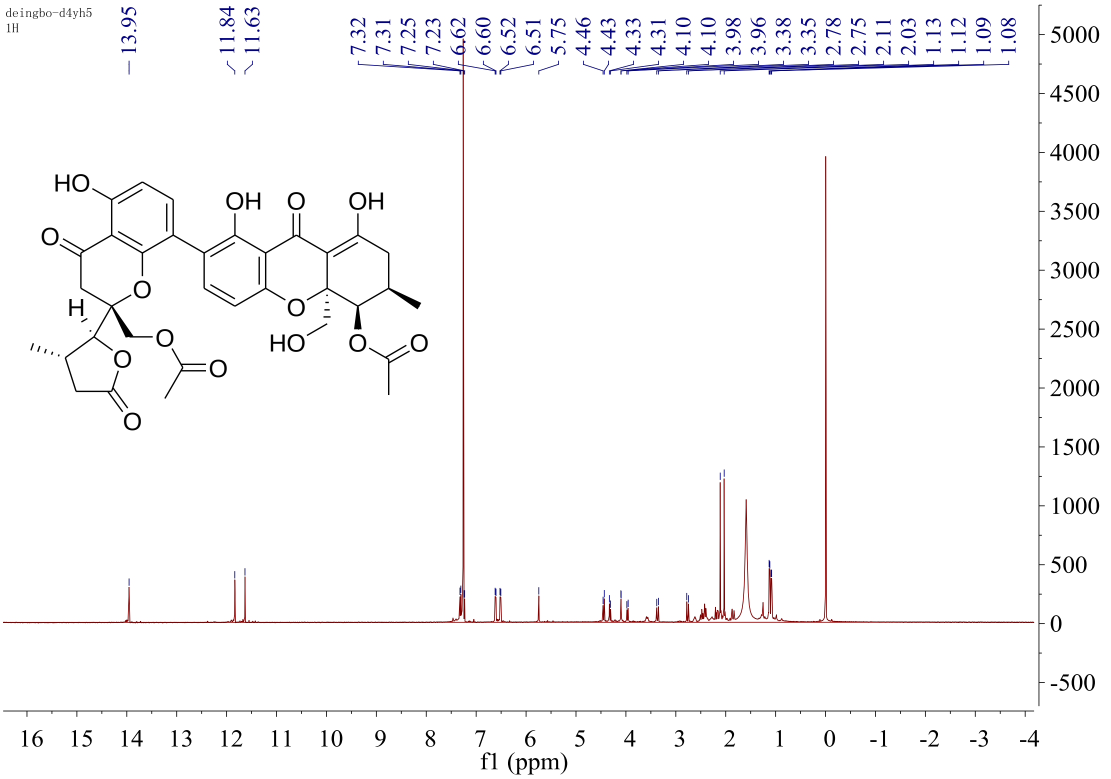

^{13}C NMR

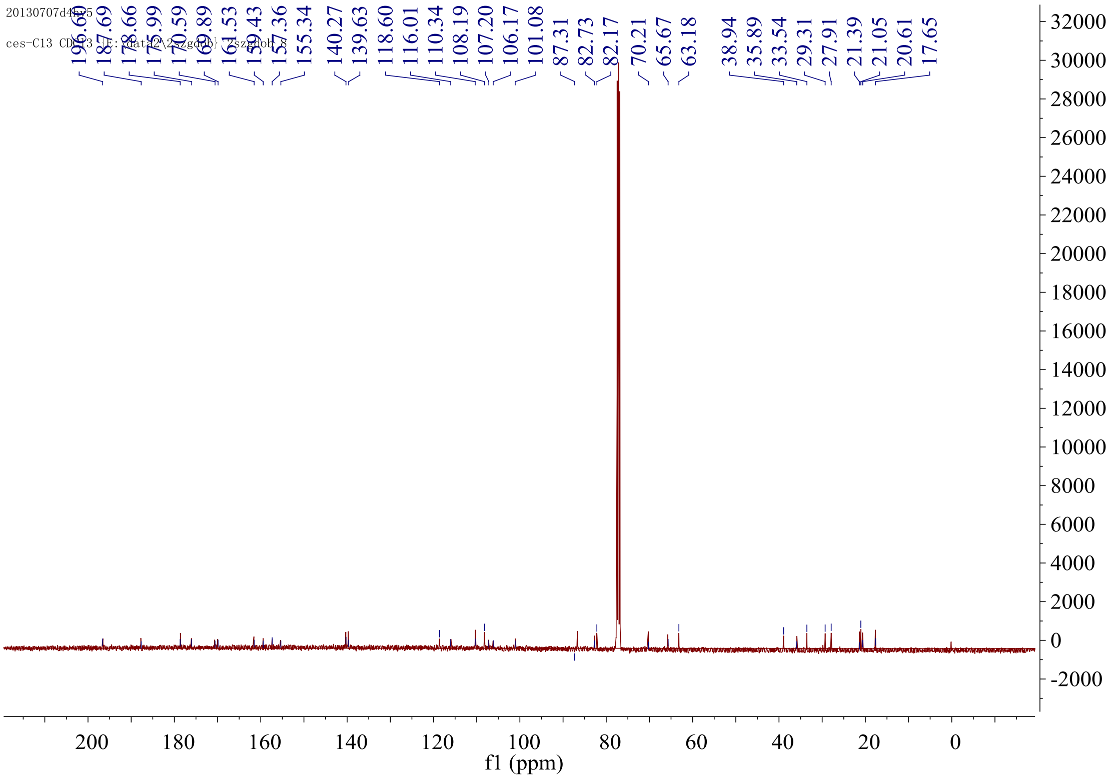

Figure S1. *Cont.*

Dept 135

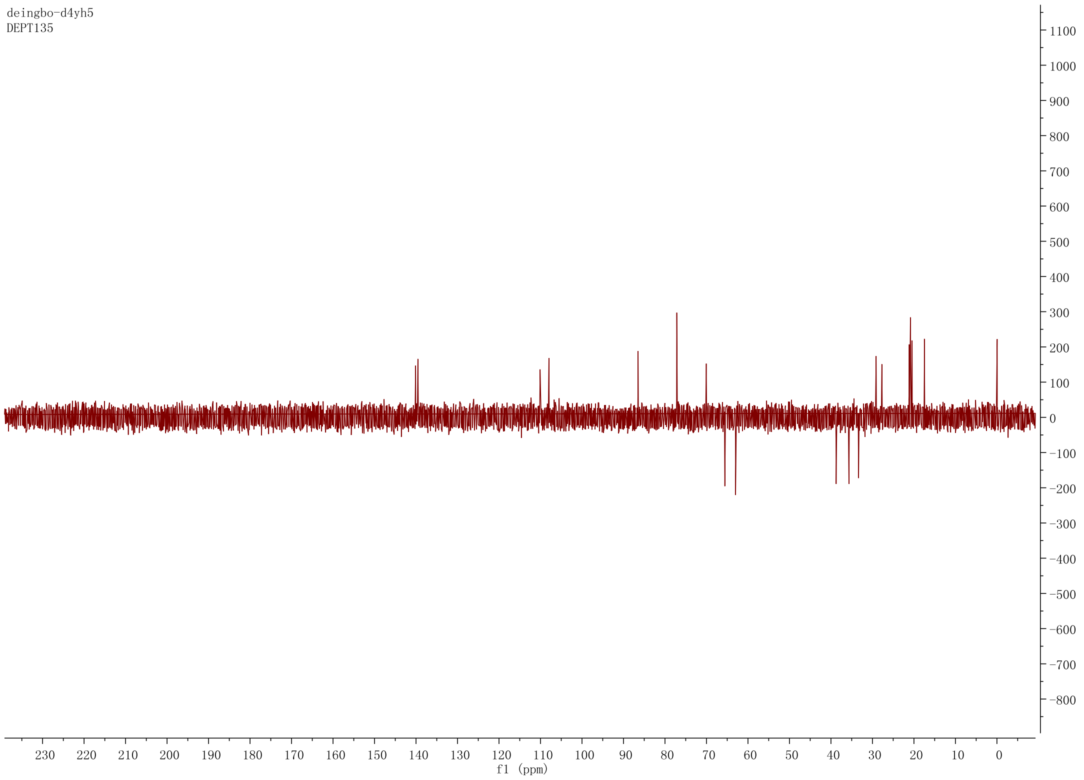

^1H-^1H COSY

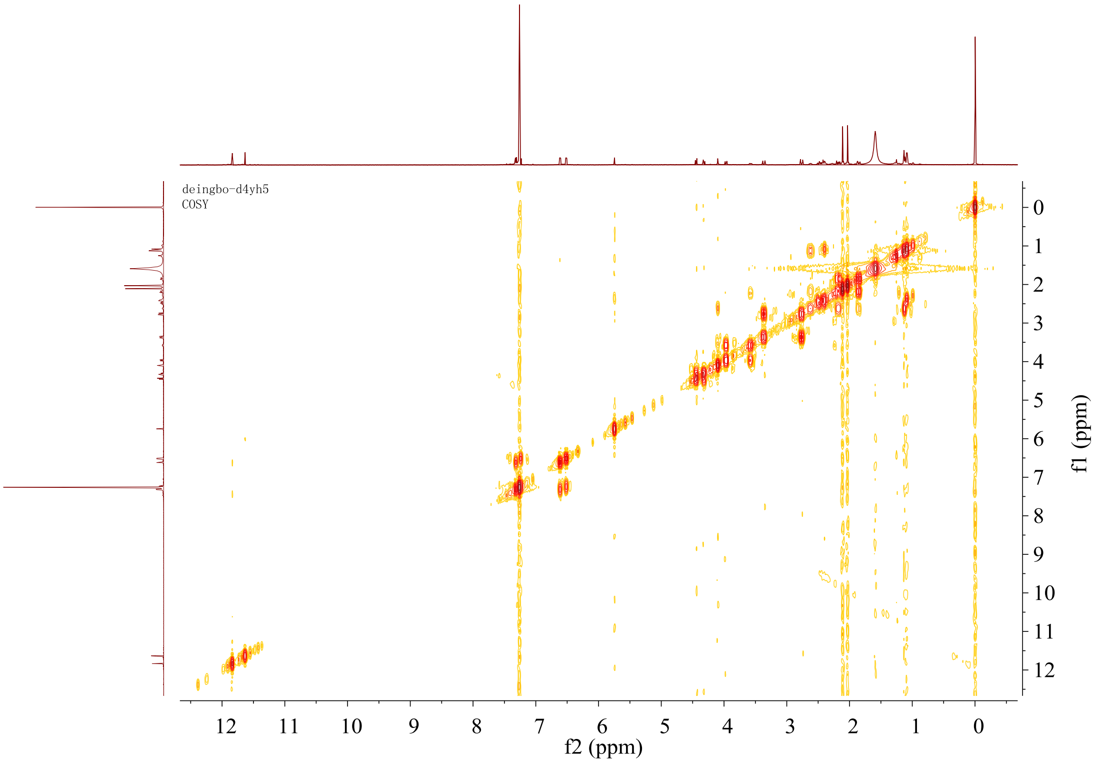

Figure S1. *Cont.*

HSQC

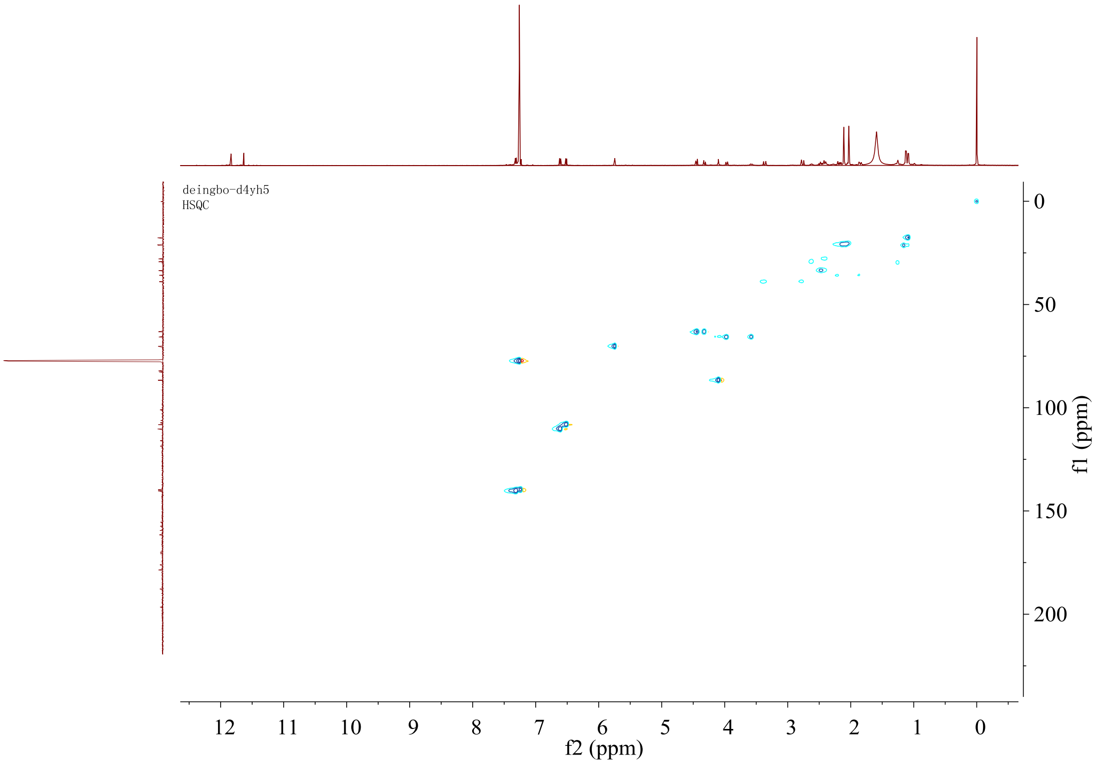

HMBC

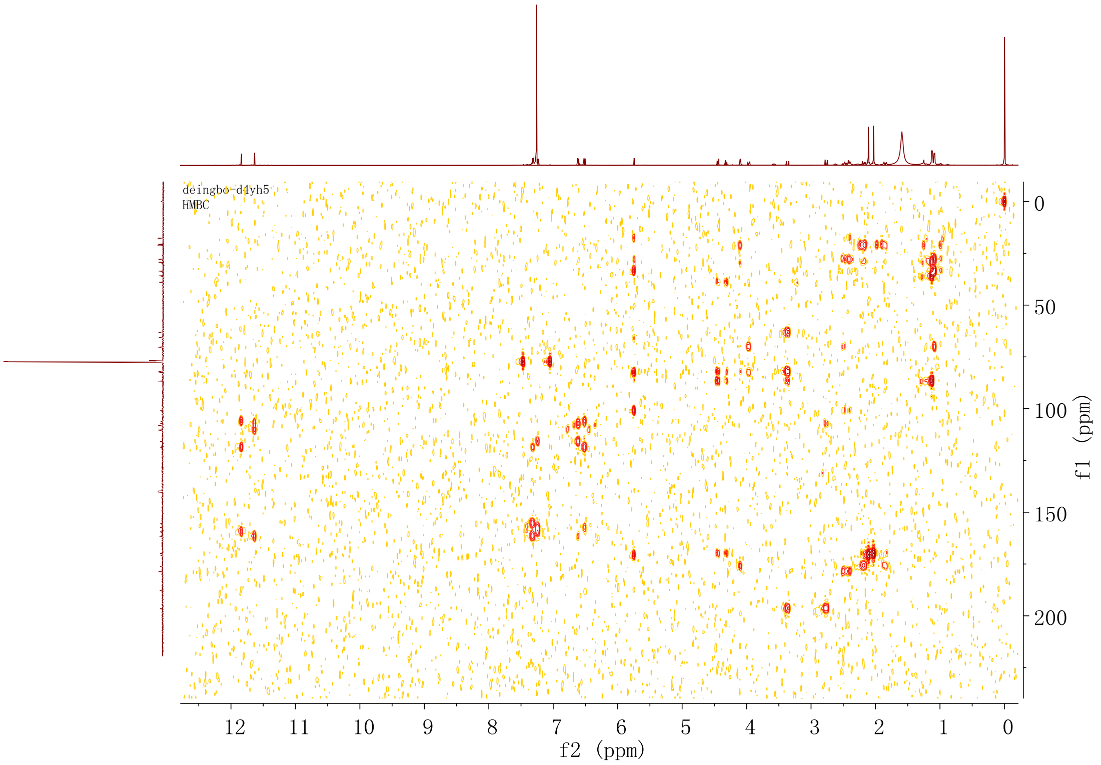

Figure S1. *Cont.*

NOE

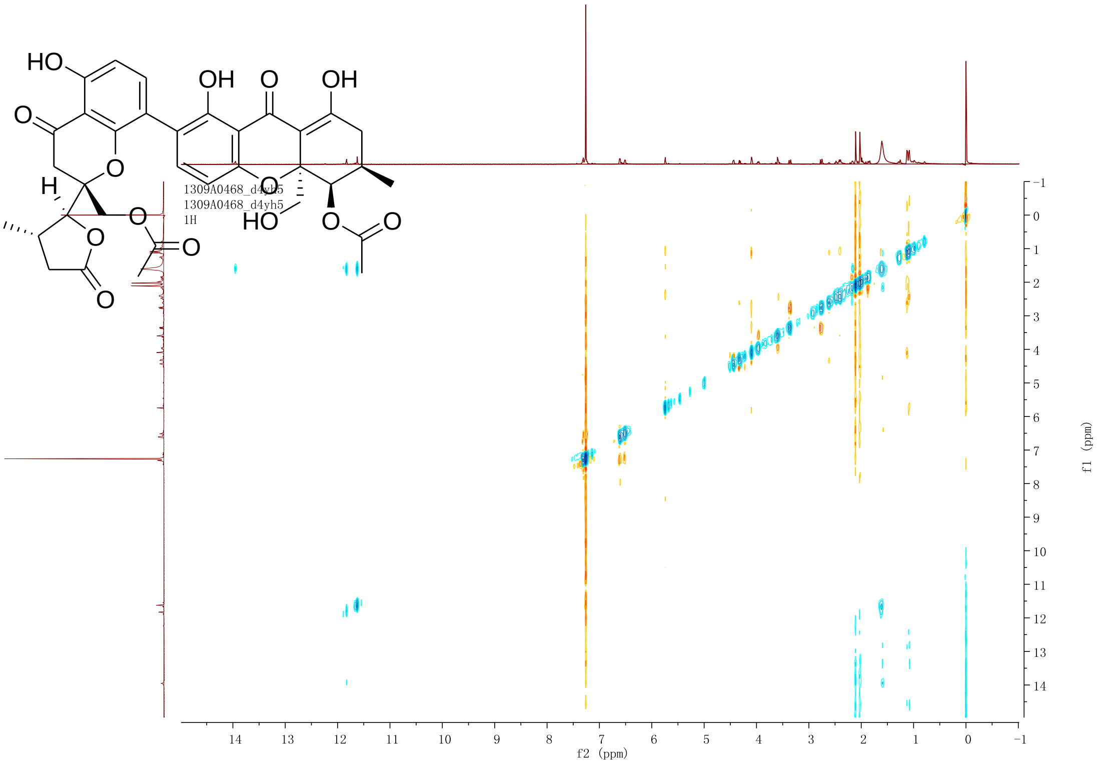

Figure S2. HRESIMS of compound **1**.

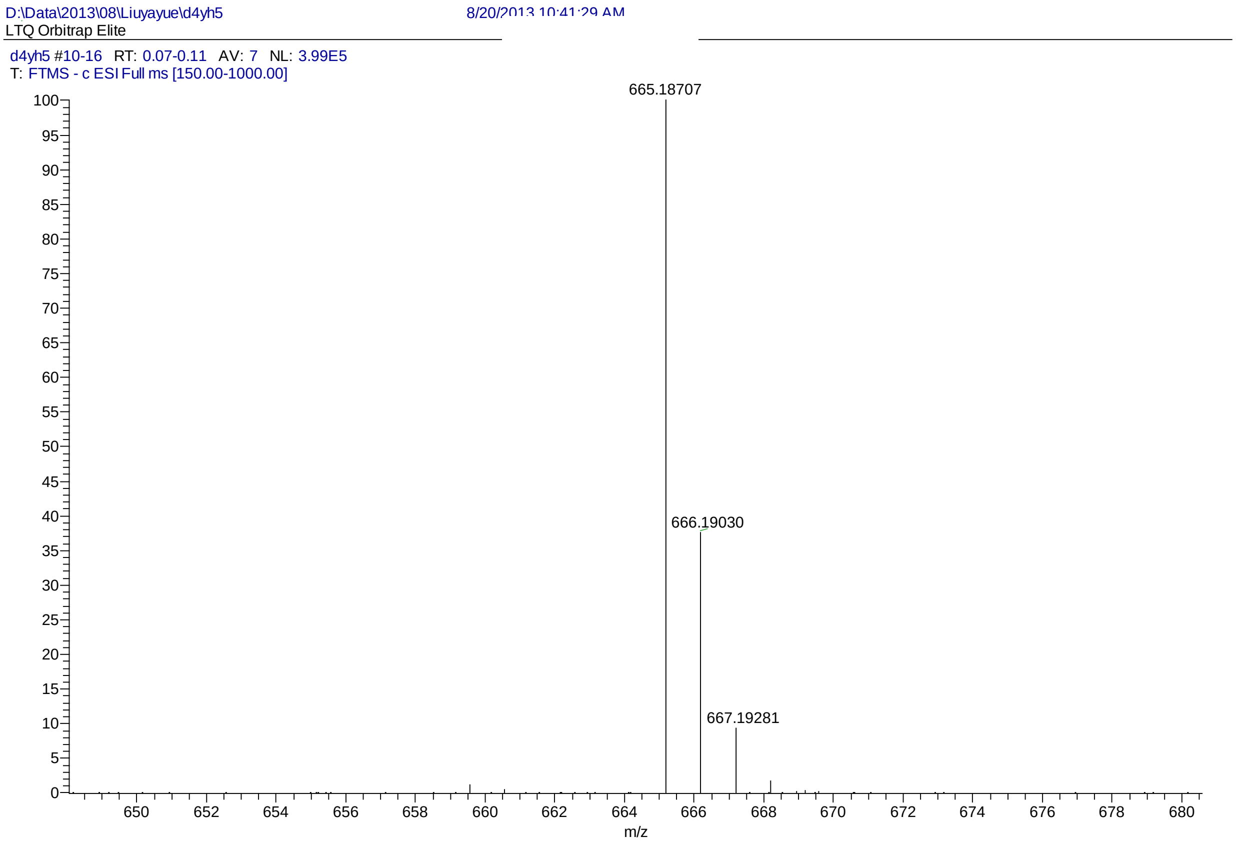

**SPECTRUM—simulation**				
m/z	Theo. Mass	Delta (ppm)	RDB equiv.	Composition
665.18707	665.18758	−0.76	18.5	C34 H33 O14

Limits:

(1) Charge: −1
(2) Nitrogen-Rule: Do not use
(3) Mass tolerance: 10.00 ppm
(4) Elements in use: ^{12}C (0–40), ^{1}H (0–60), ^{16}O (0–15)

Figure S3. IR of compound **1**.

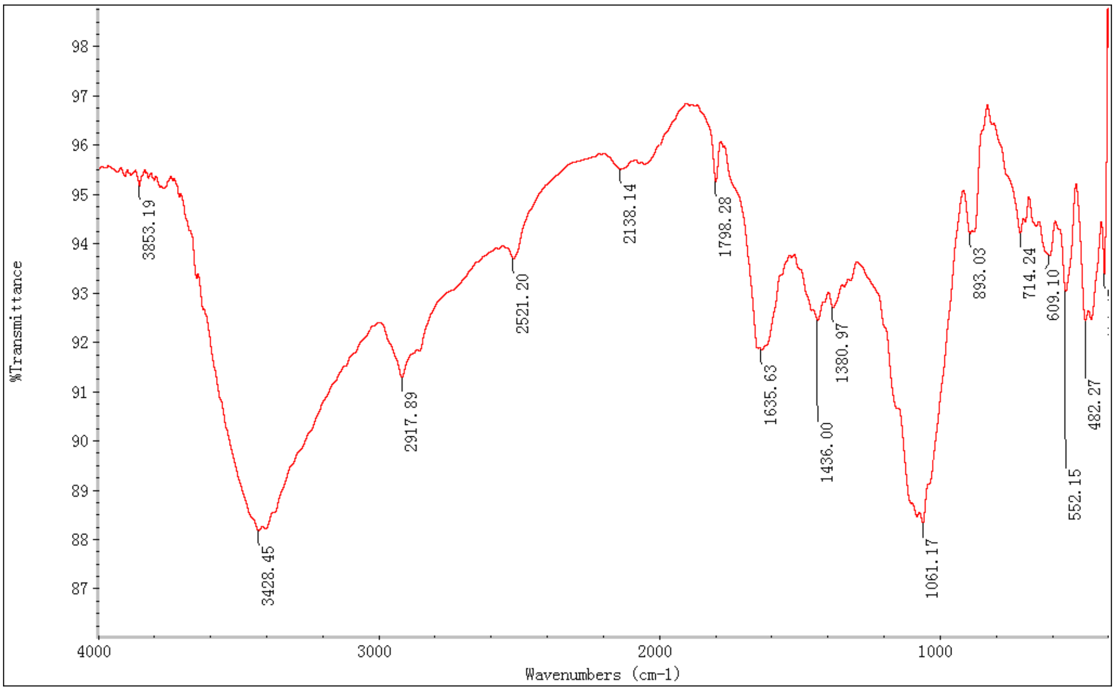

Figure S4. UV of compound **1**.

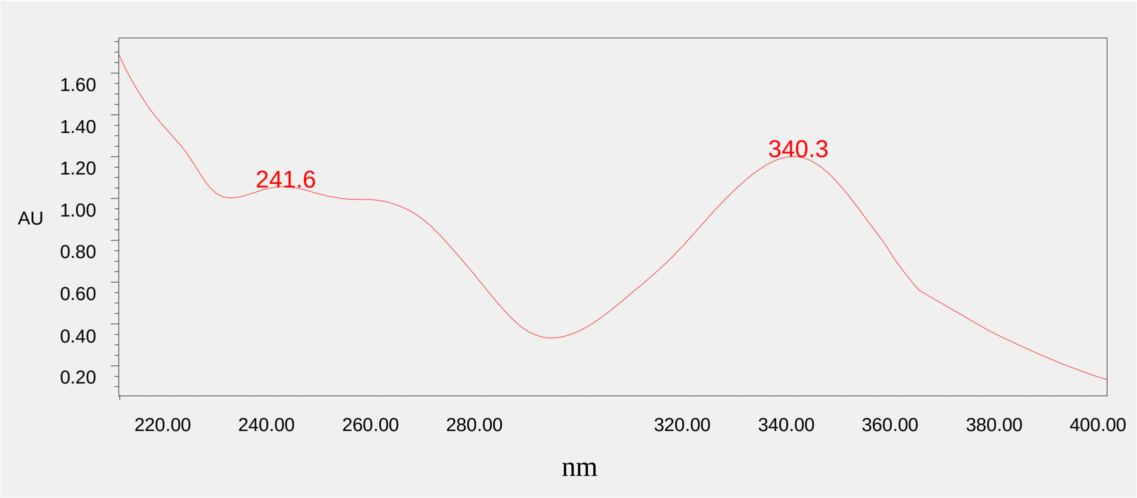

Figure S5. ^{1}H, ^{13}C, ^{1}H-^{1}H COSY, HSQC, HMBC, NOE of compound **2**.

^{1}H NMR

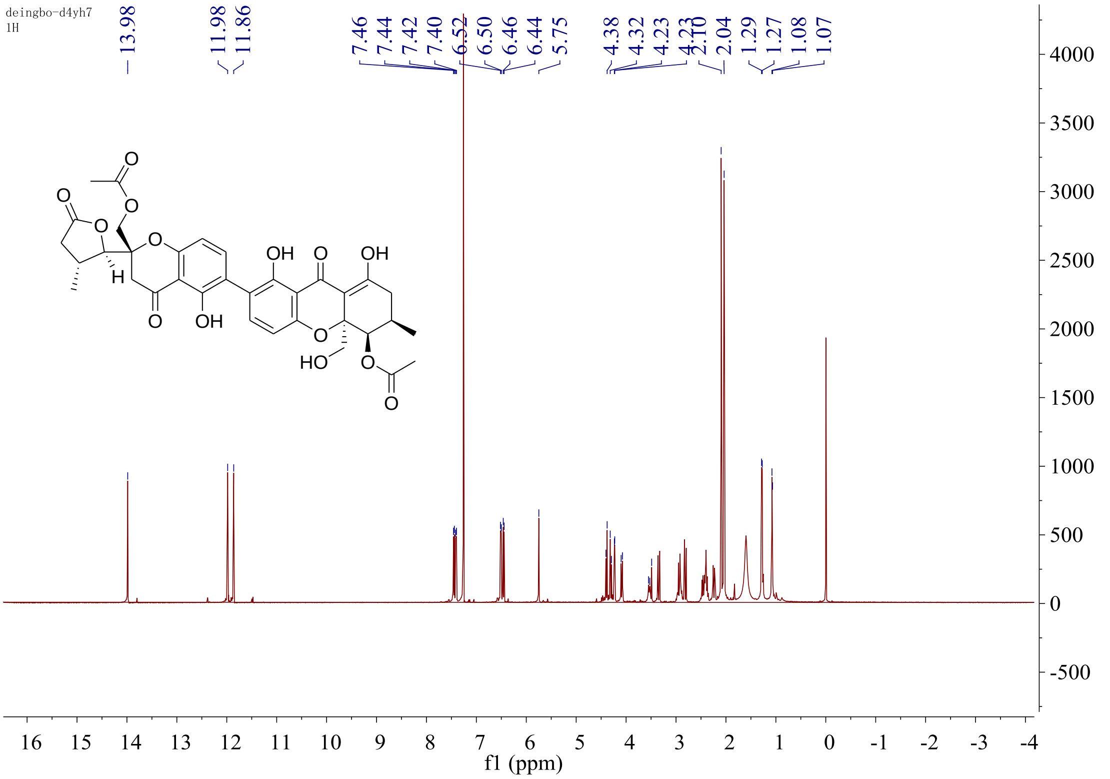

^{13}C NMR

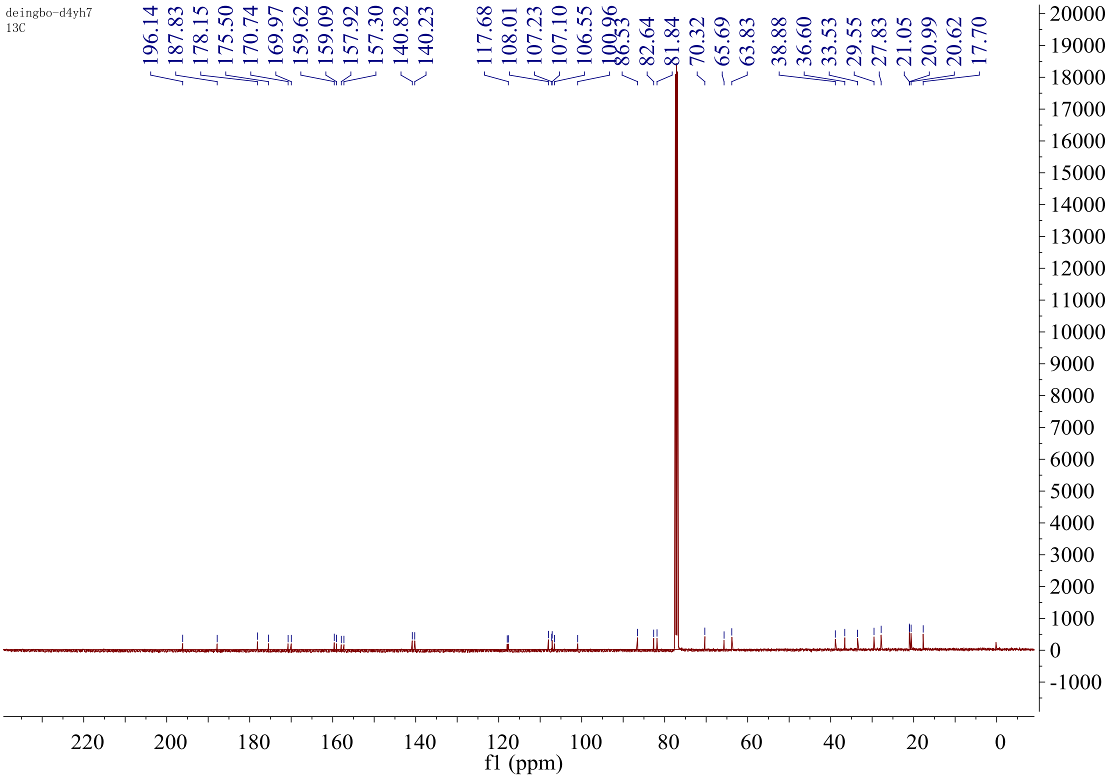

Figure S5. *Cont.*

^1H-^1H COSY

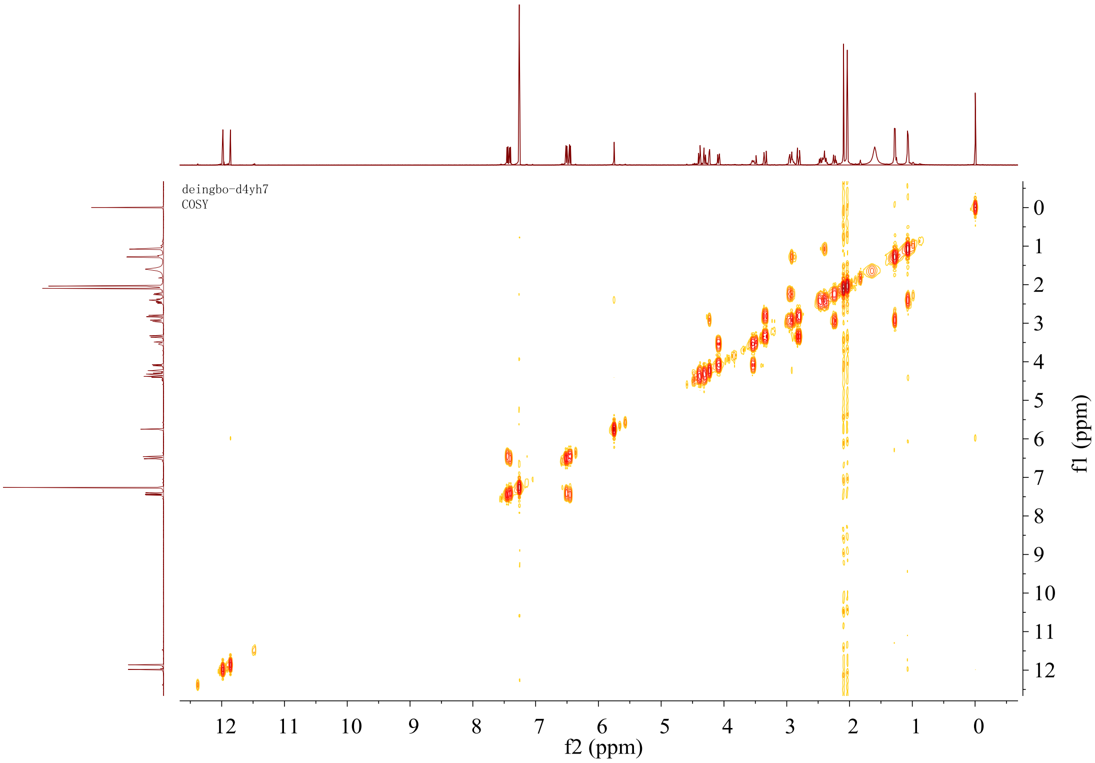

HSQC

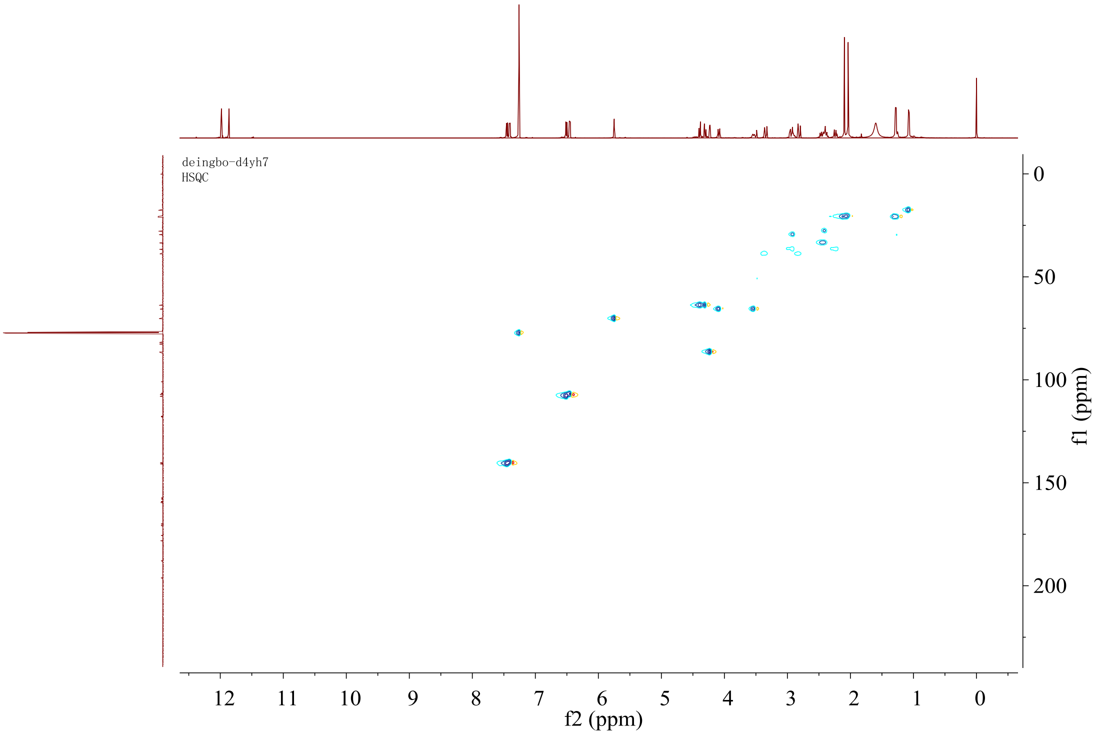

Figure S5. *Cont.*

HMBC

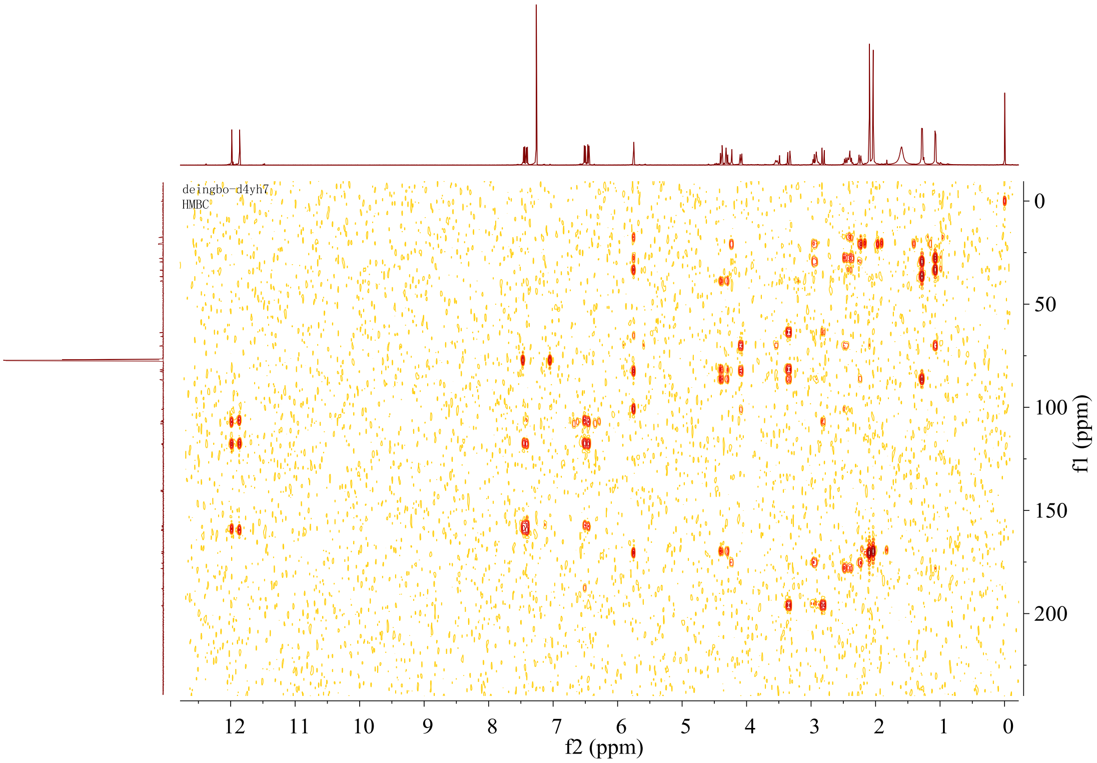

NOE

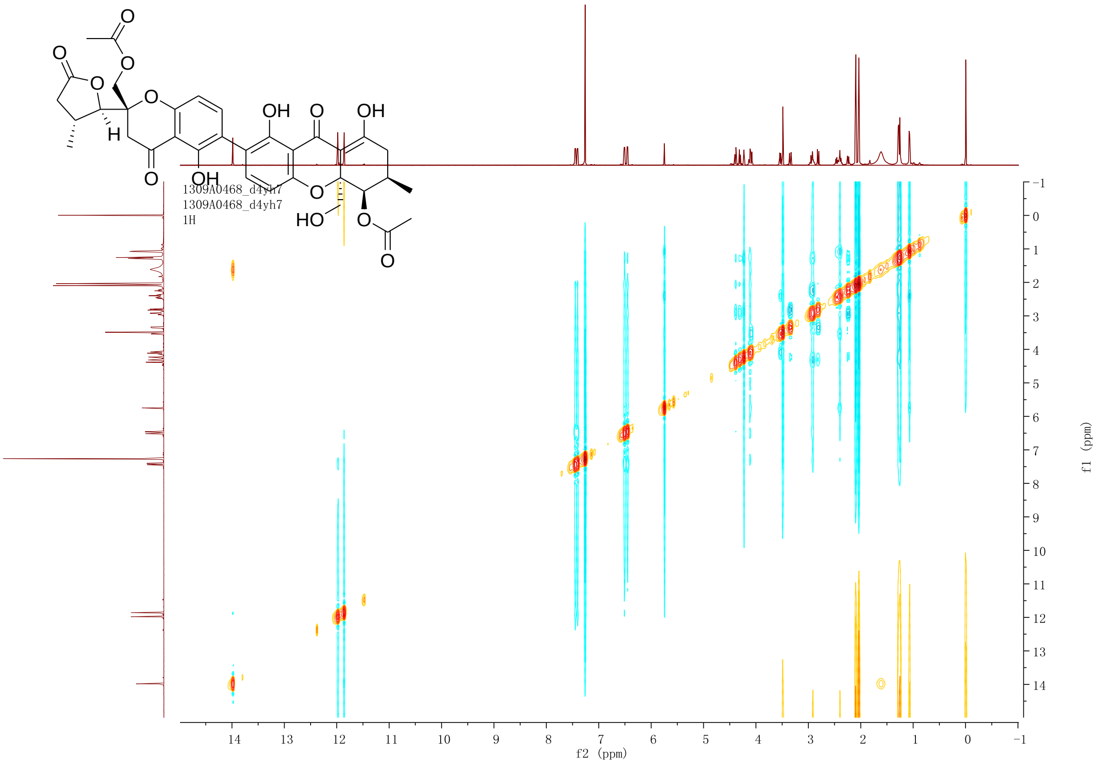

Figure S6. HRESIMS of compound **2**.

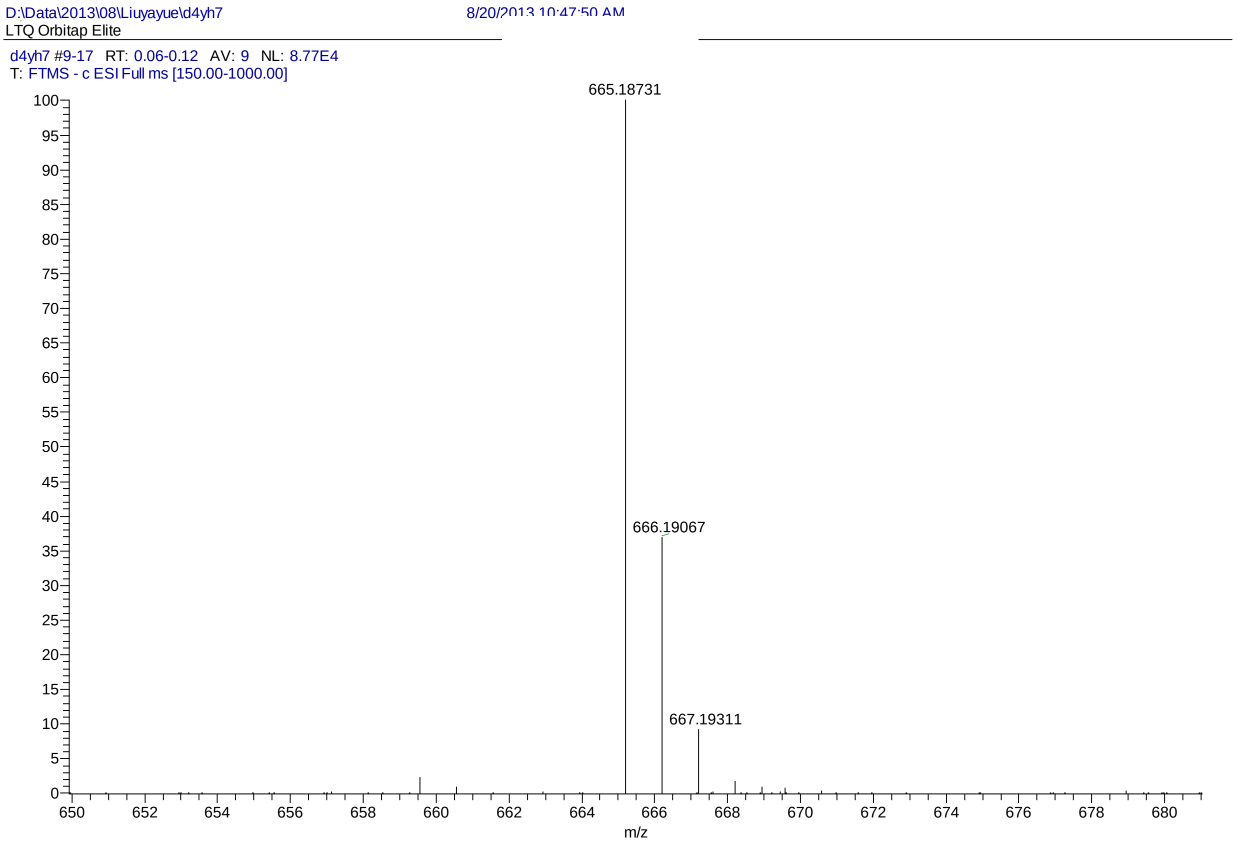

**SPECTRUM—simulation**				
m/z	Theo. Mass	Delta (ppm)	RDB equiv.	Composition
665.18731	665.18758	−0.40	18.5	C34 H33 O14

Limits:

(1) Charge: −1
(2) Nitrogen-Rule: Do not use
(3) Mass tolerance: 10.00 ppm
(4) Elements in use: ^{12}C (0–40), ^{1}H (0–60), ^{16}O (0–15)

Figure S7. IR of compound **2**.

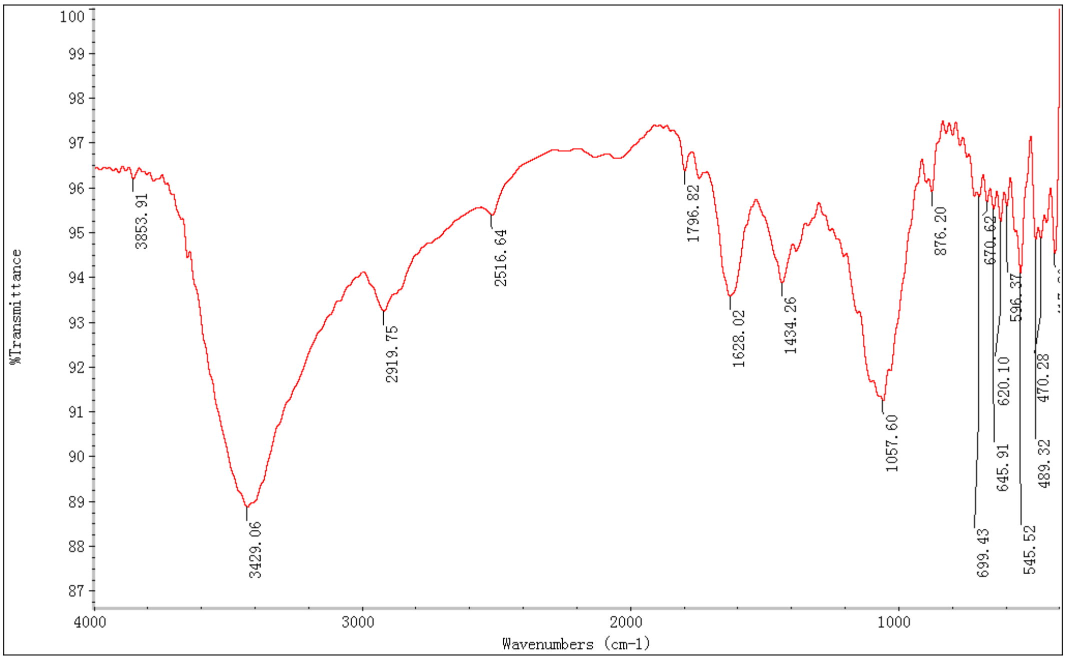

Figure S8. UV of compound **2**.

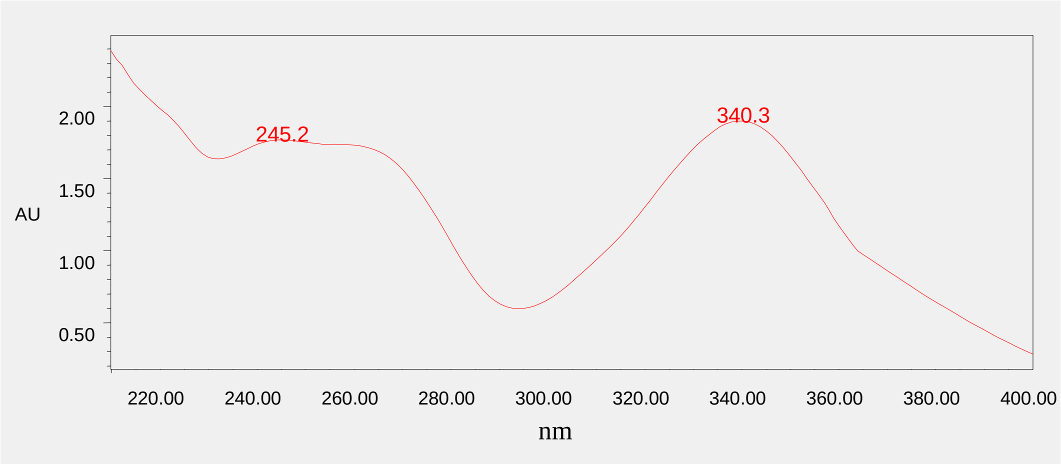

Figure S9. ^{1}H, ^{13}C, Dept 135, ^{1}H-^{1}H COSY, HSQC, HMBC, NOE of compound **3**.

^{1}H NMR

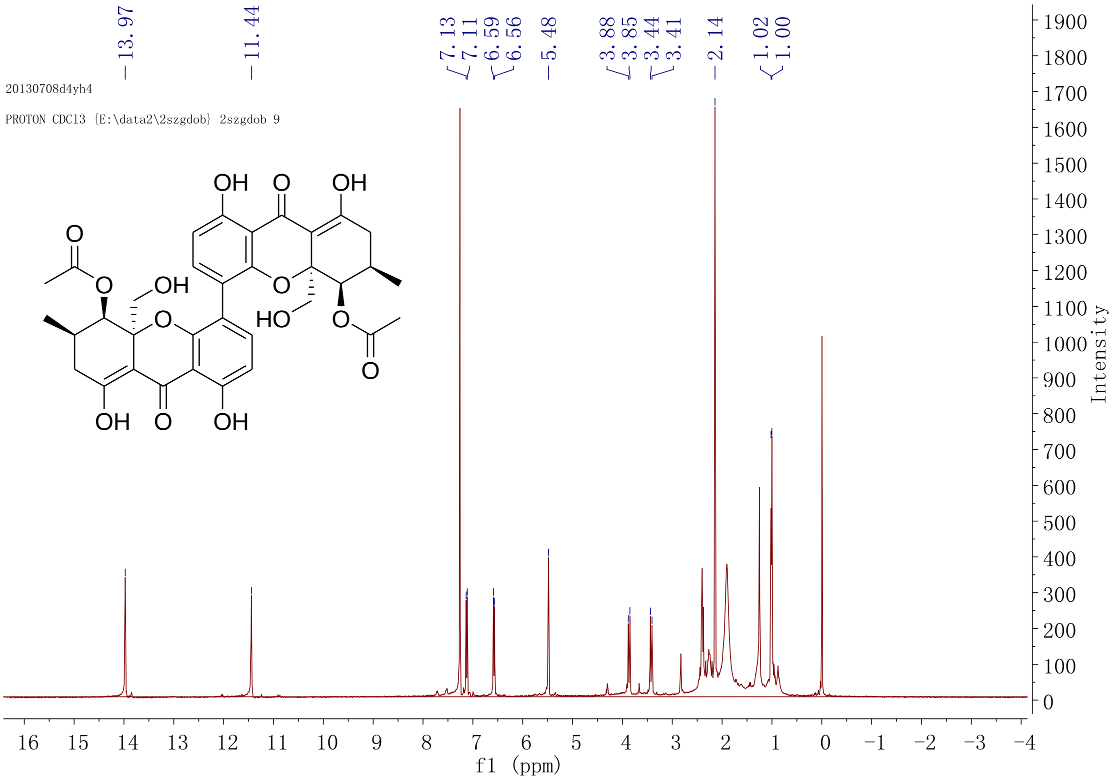

^{13}C NMR

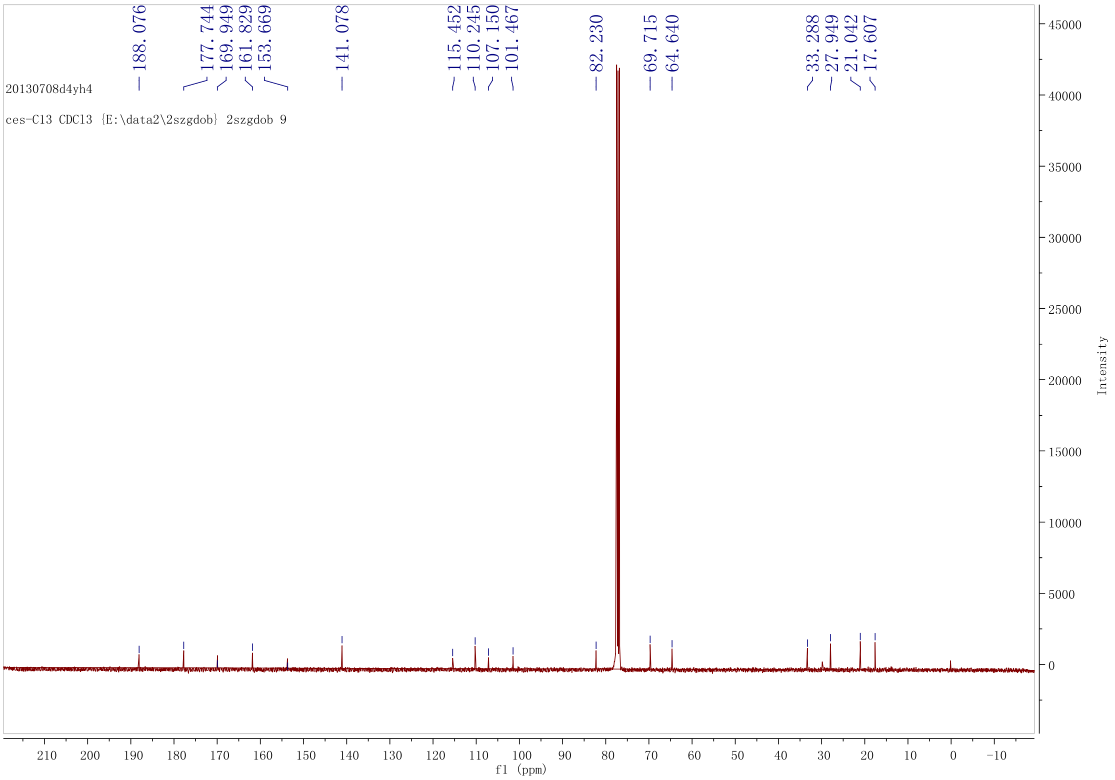

Figure S9. *Cont.*

Dept 135

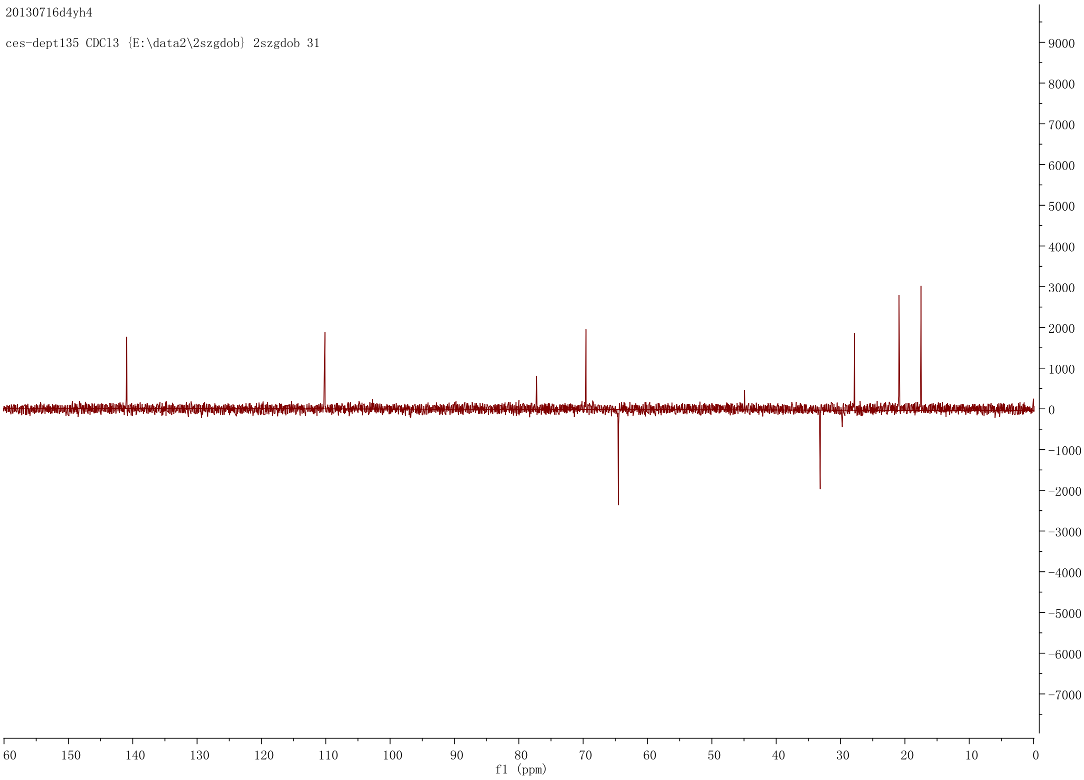

^1H-^1H COSY

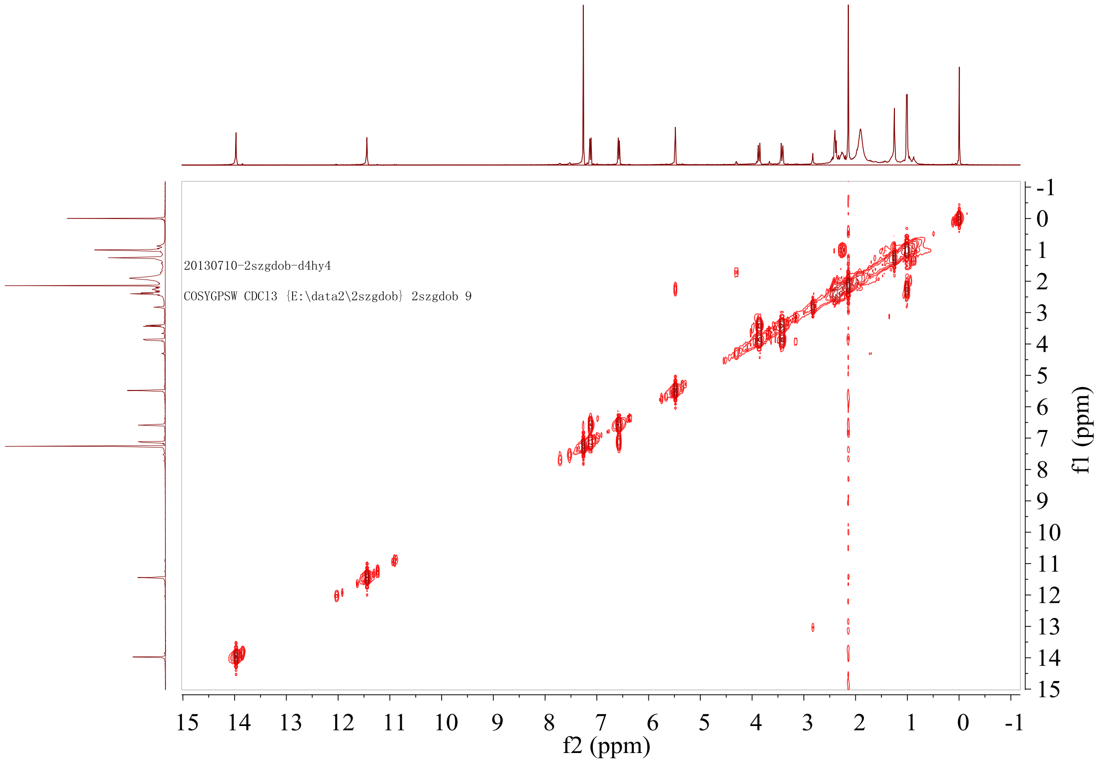

Figure S9. *Cont.*

HSQC

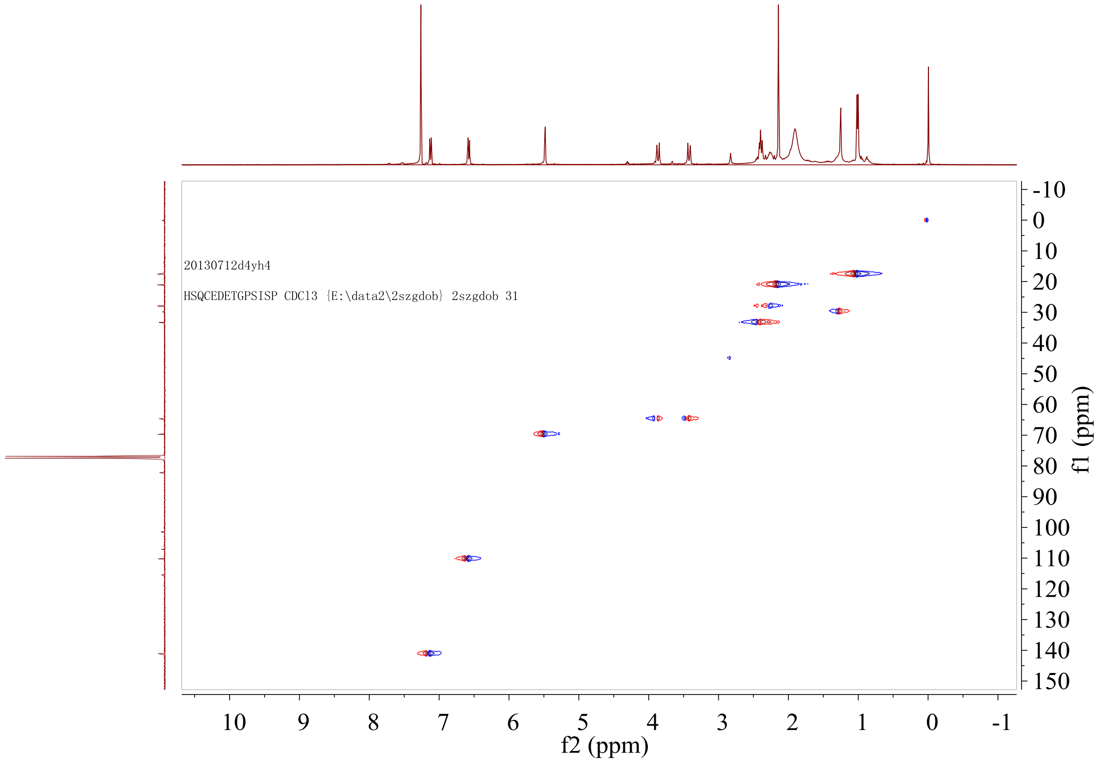

HMBC

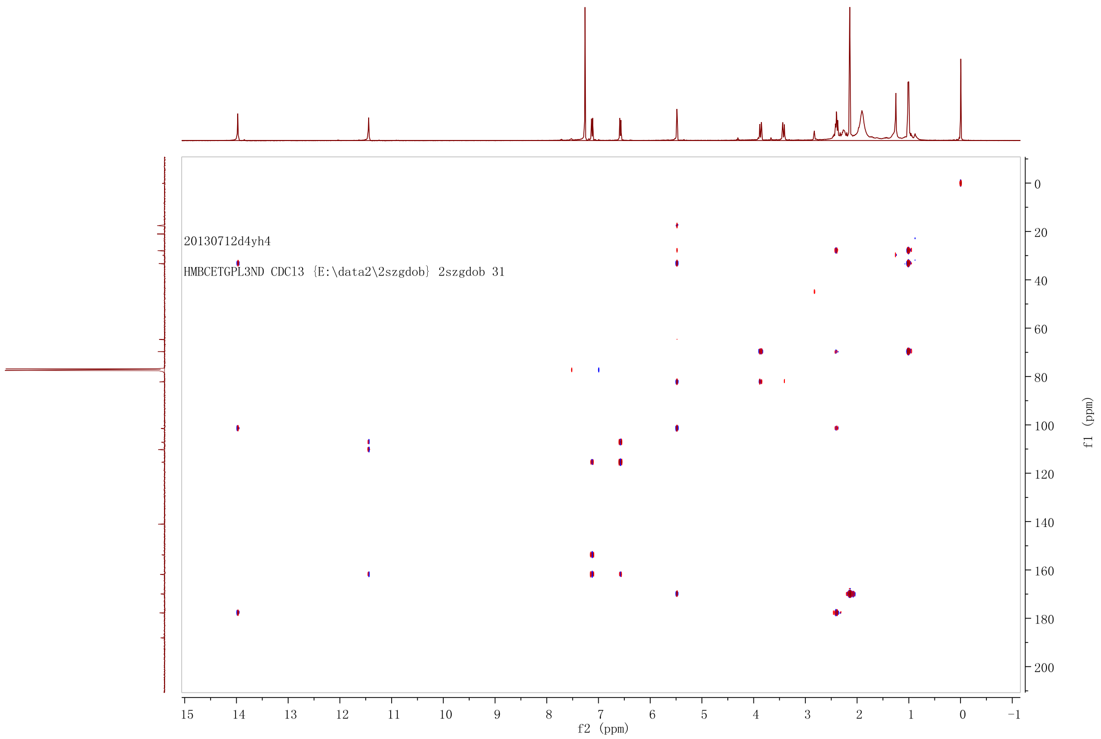

Figure S9. *Cont.*

NOE

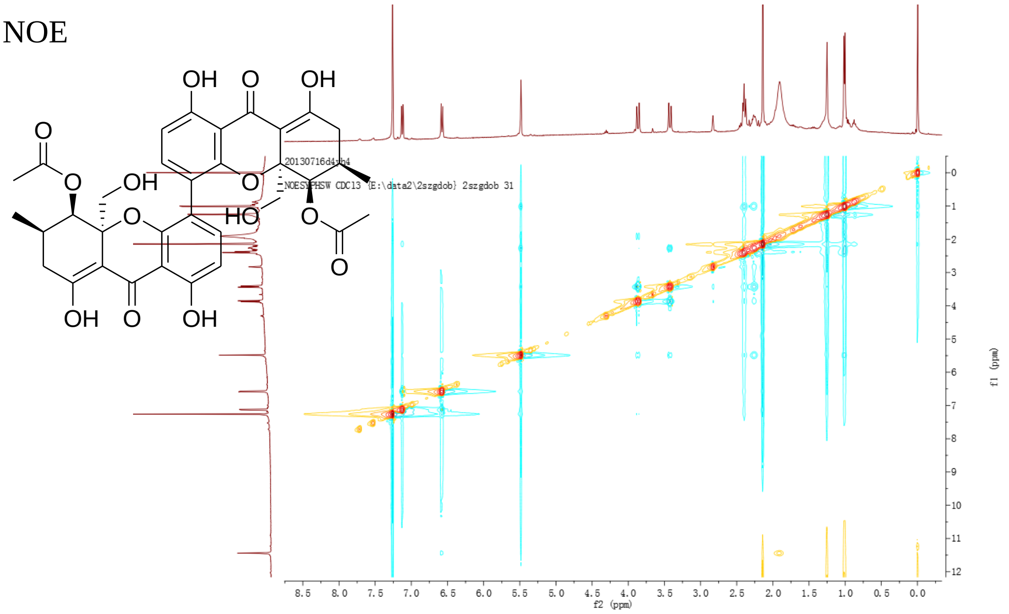

Figure S10. HRESIMS of compound **3**.

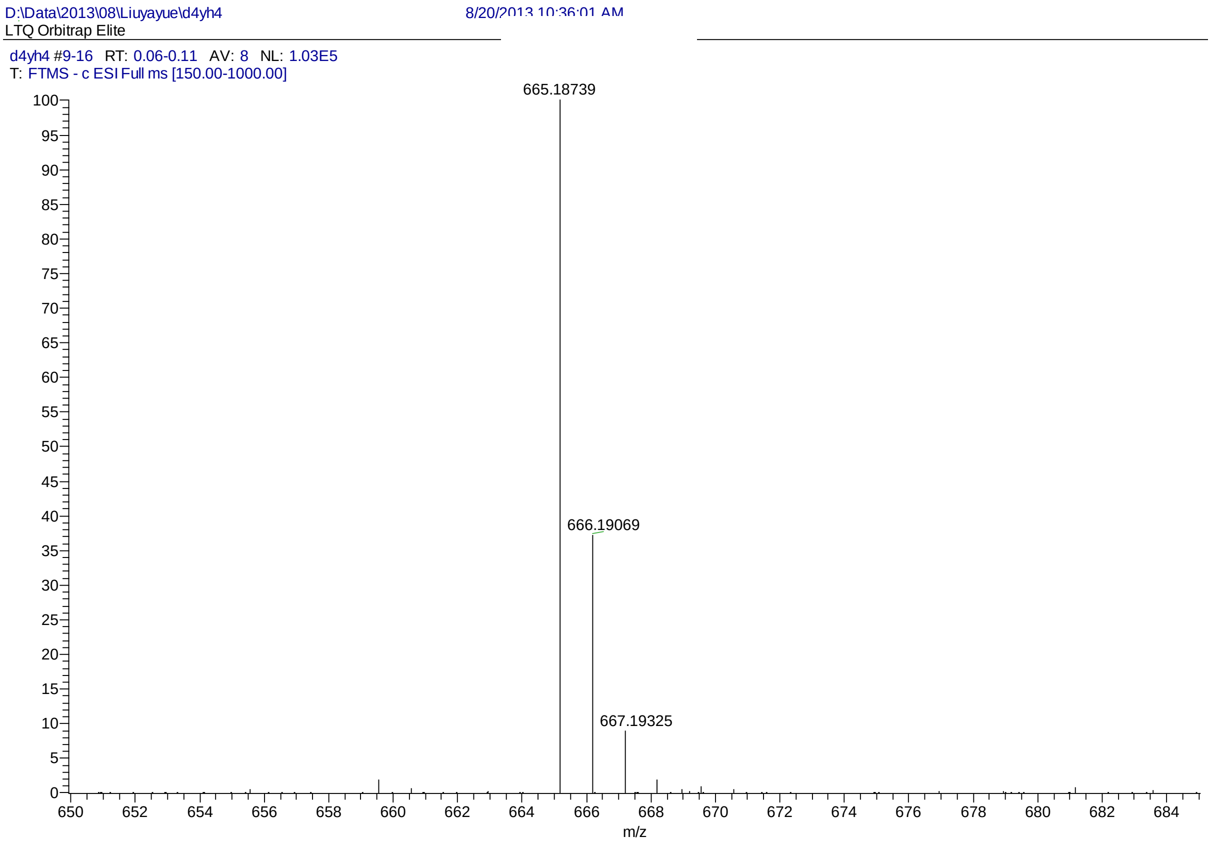

SPECTRUM—simulation				
m/z	Theo. Mass	Delta (ppm)	RDB equiv.	Composition
665.18739	665.18758	−0.28	18.5	C34 H33 O14

Limits:

(1) Charge: −1
(2) Nitrogen-Rule: Do not use
(3) Mass tolerance: 10.00 ppm
(4) Elements in use: ^{12}C (0–40), ^{1}H (0–60), ^{16}O (0–15)

Figure S11. IR of compound **3**.

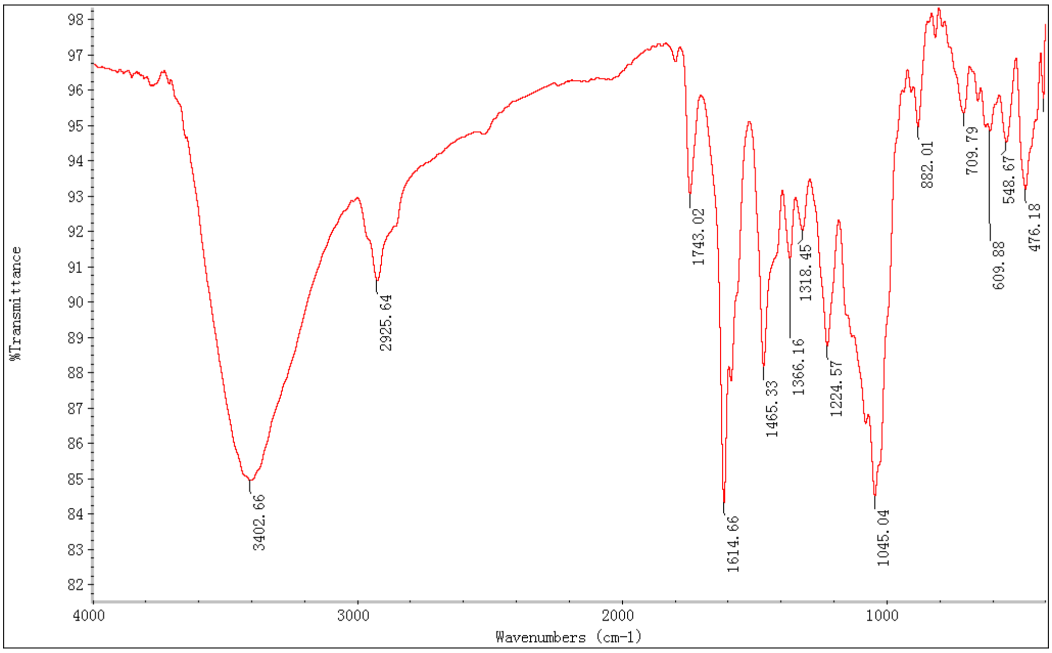

Figure S12. UV of compound **3**.

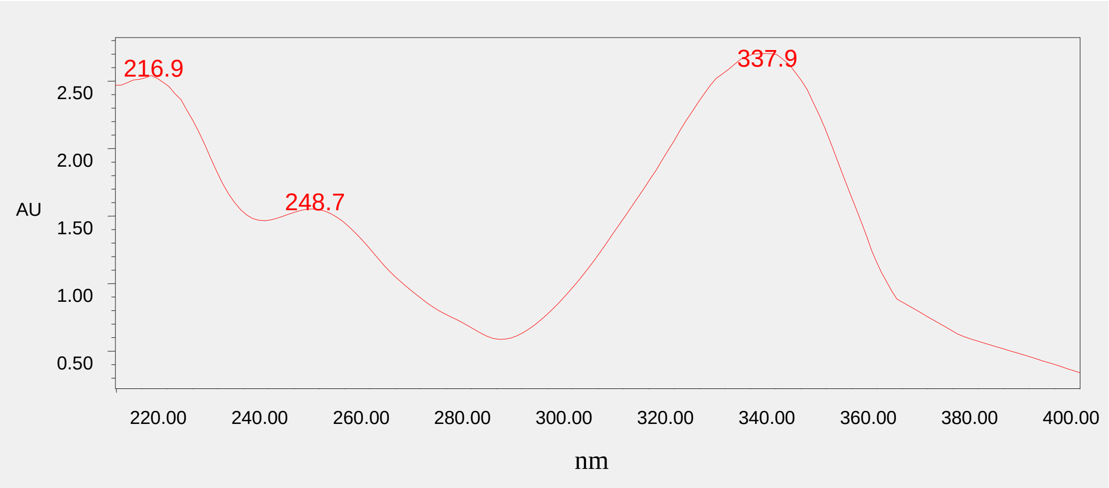

Figure S13. ^{1}H, ^{13}C, ^{1}H-^{1}H COSY, HSQC, HMBC, NOE of compound **4**.

^{1}H

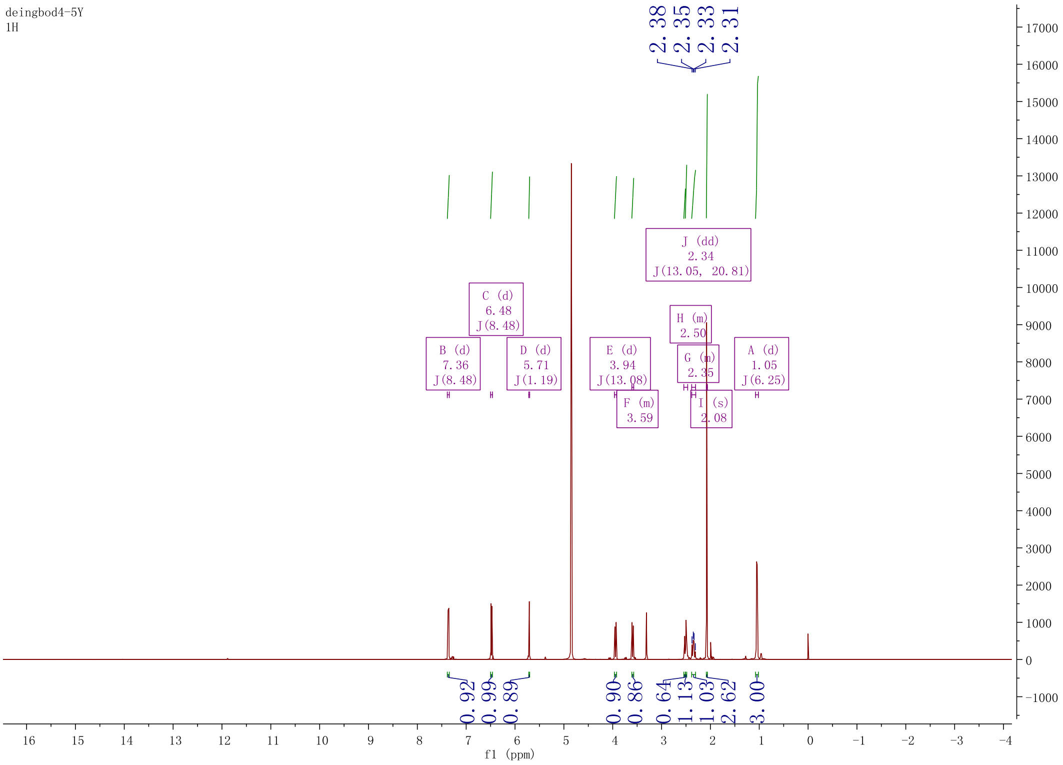

^{13}C

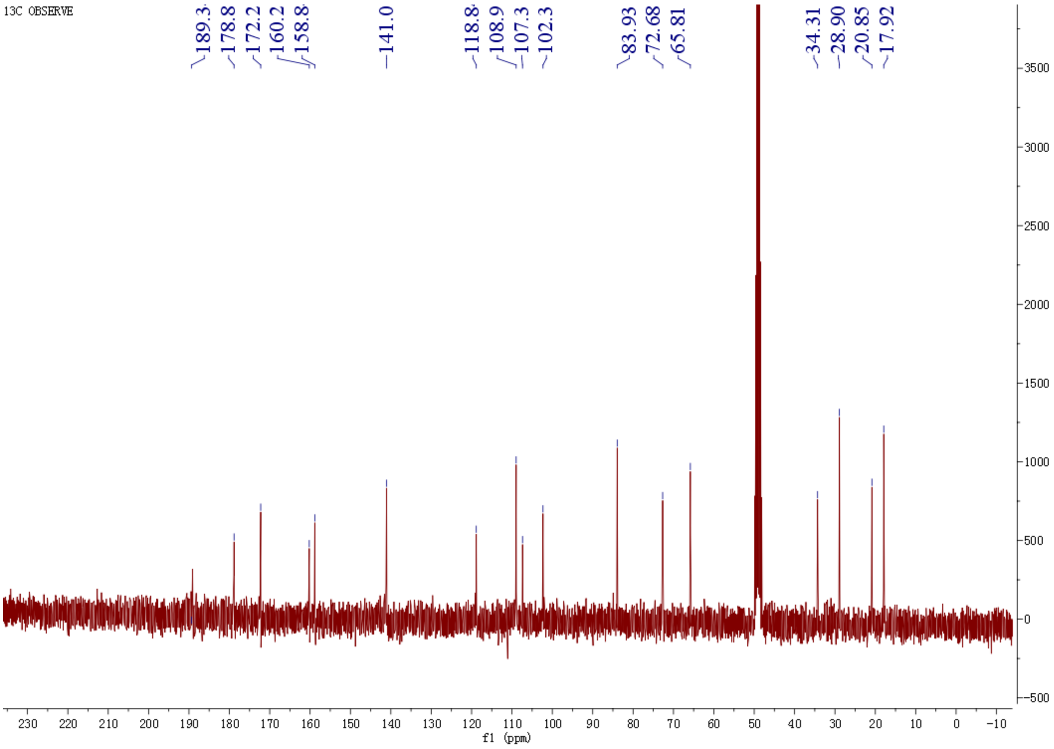

Figure S13. *Cont.*

^{1}H-^{1}H COSY

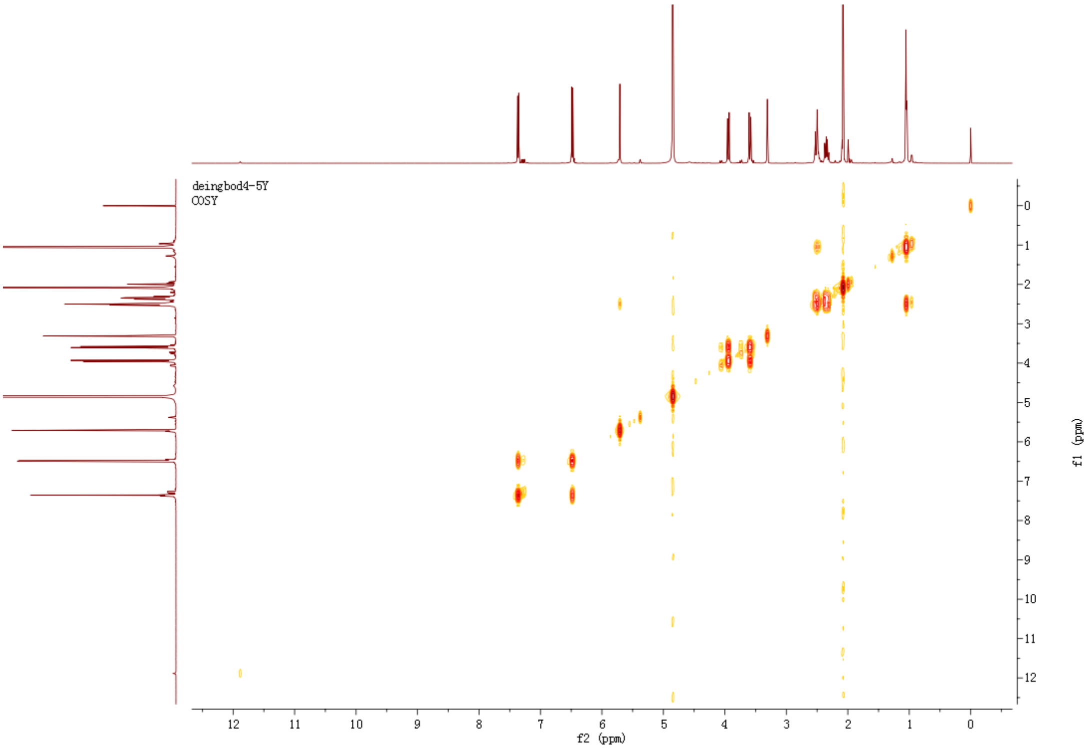

HSQC

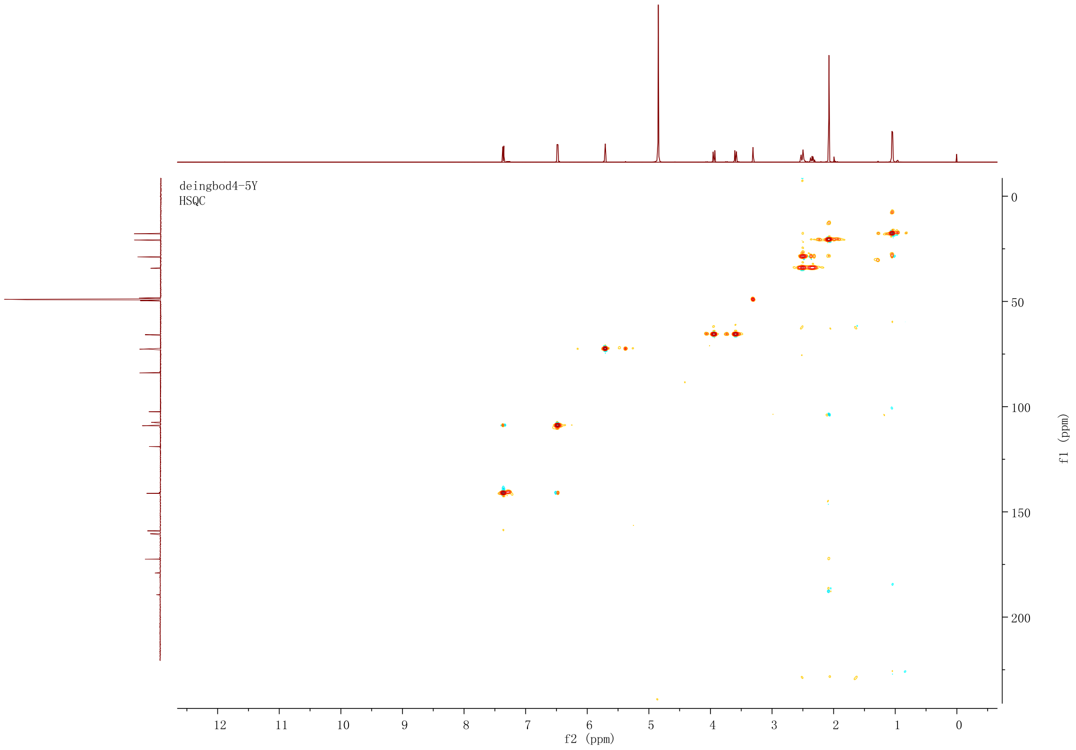

Figure S13. *Cont.*

HMBC

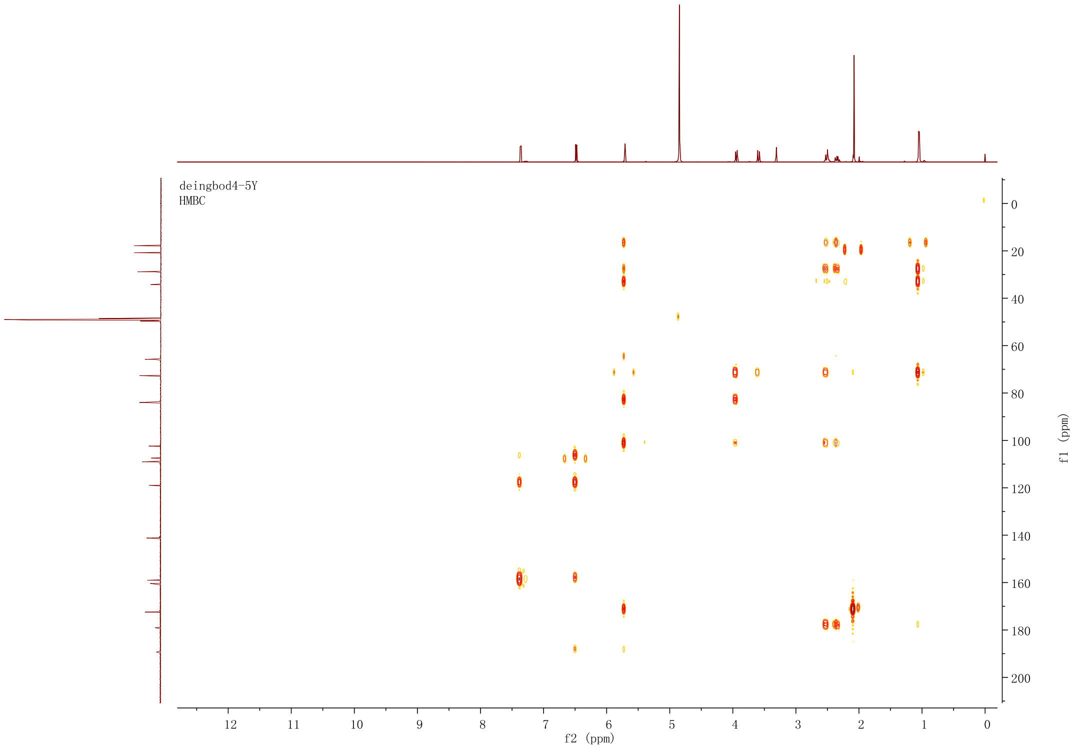

NOE

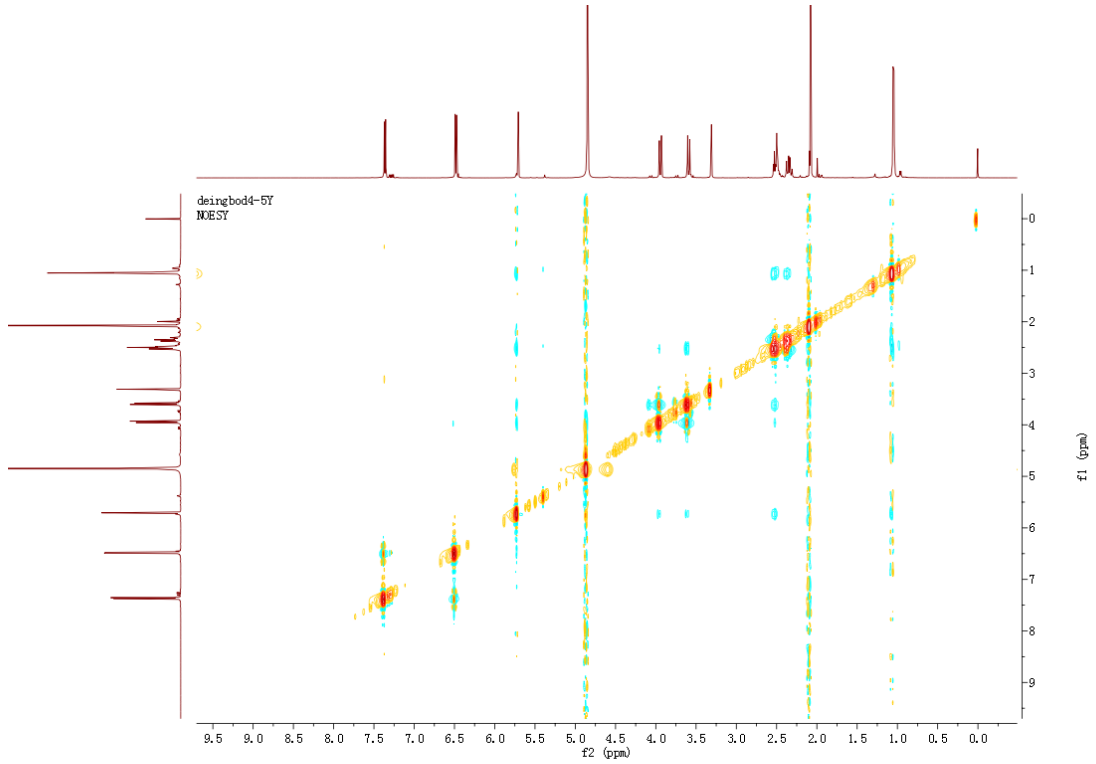

Figure S14. ^{1}H, ^{13}C, ^{1}H-^{1}H COSY, HSQC, HMBC, NOE of compound **5**.

^{1}H

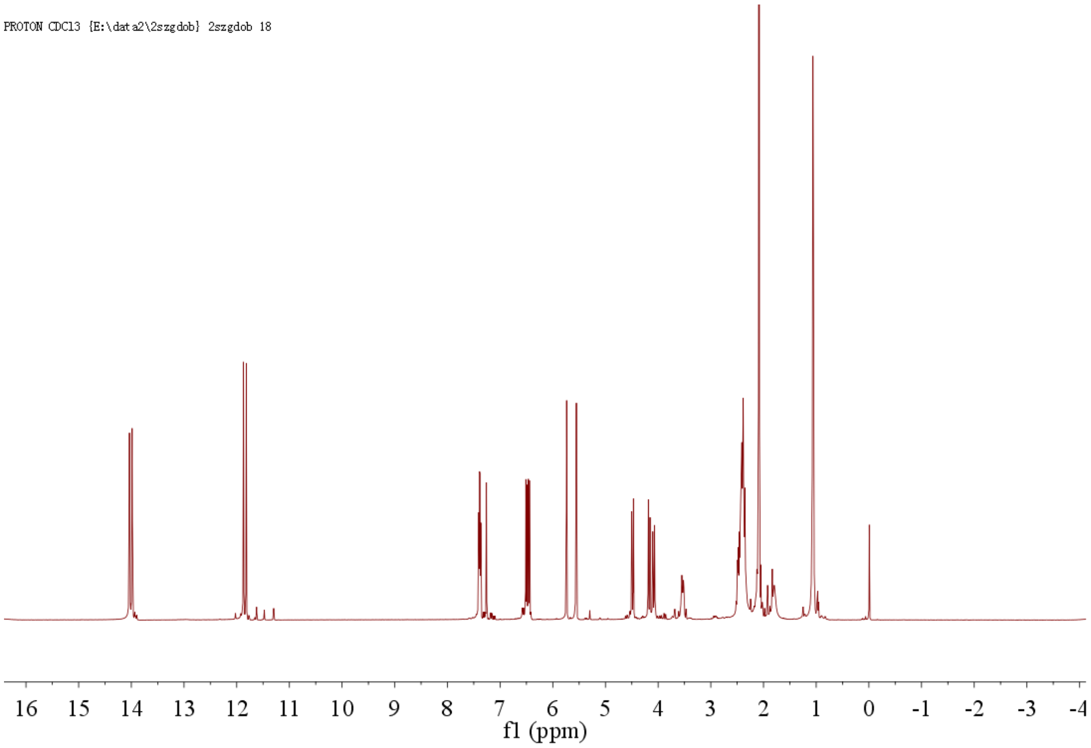

^{13}C

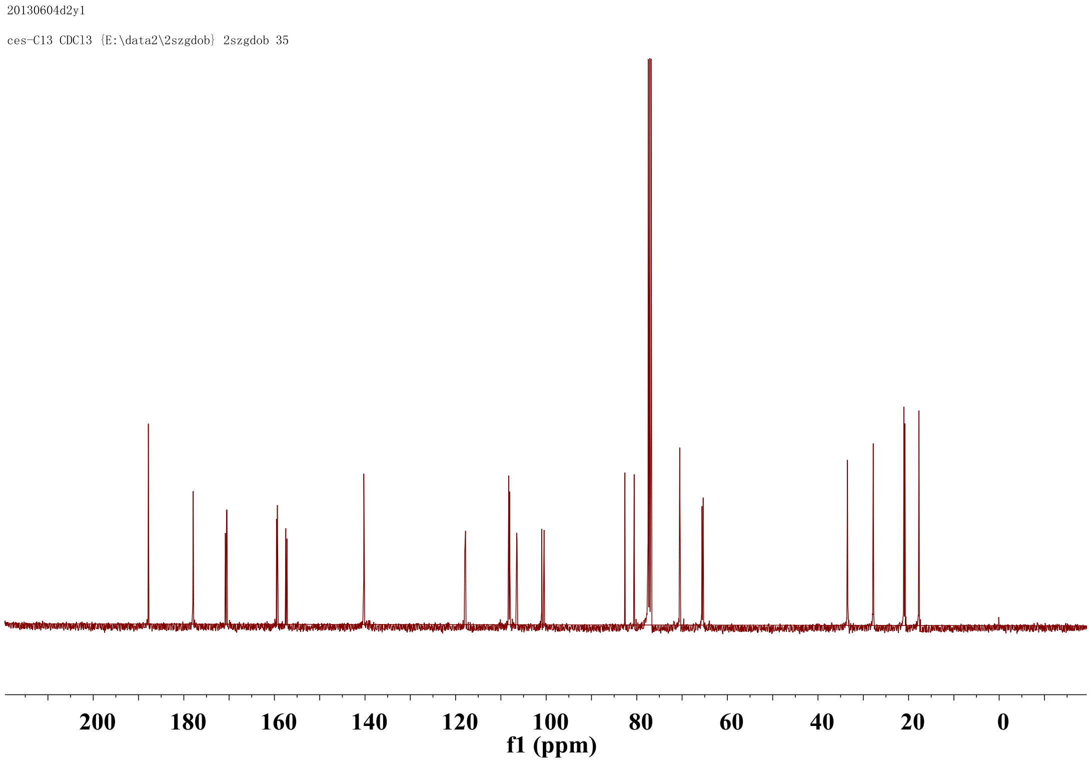

Figure S14. *Cont.*

^1H-^1H COSY

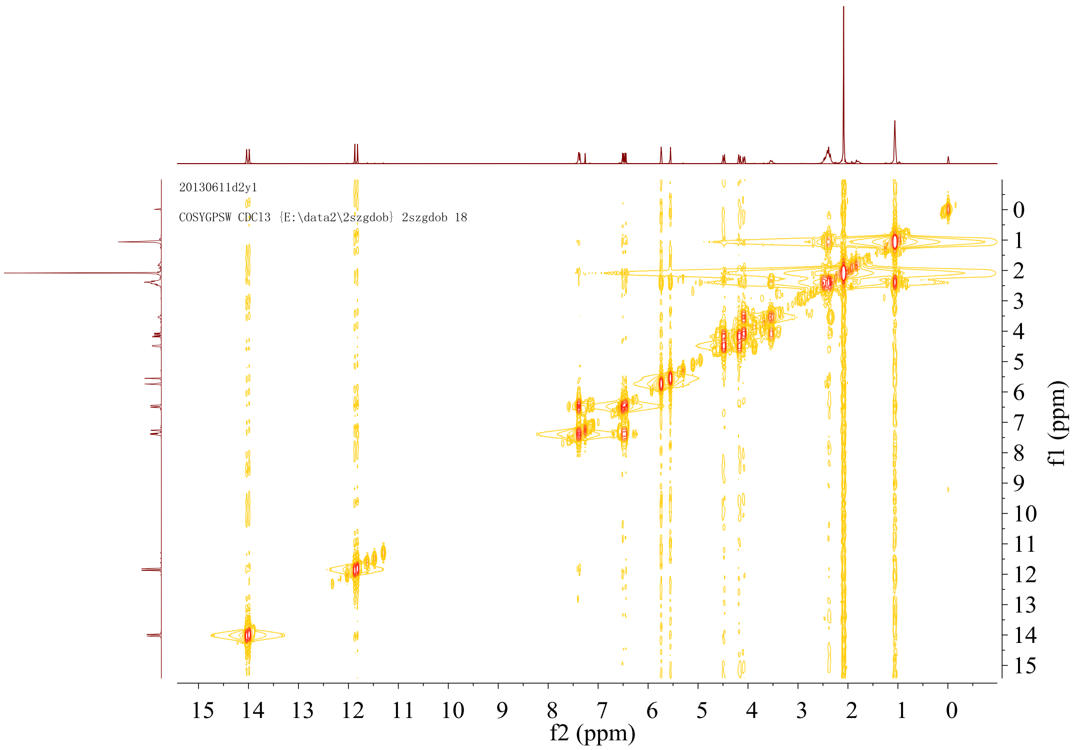

HSQC

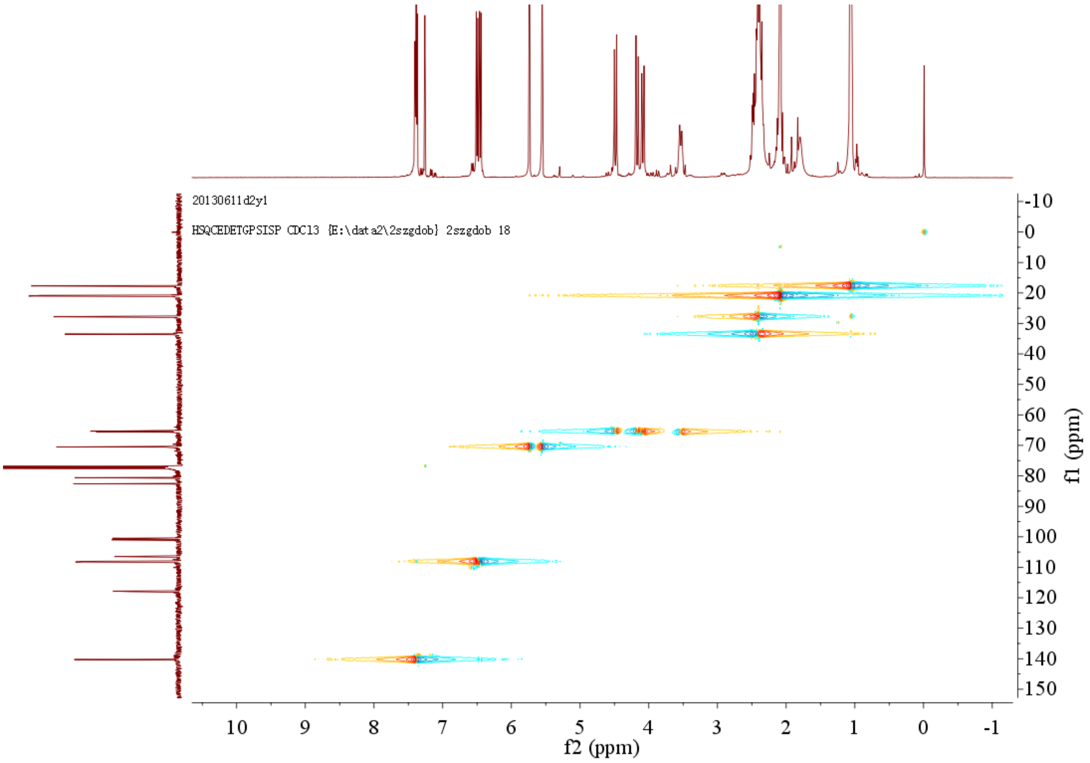

Figure S14. *Cont.*

HMBC

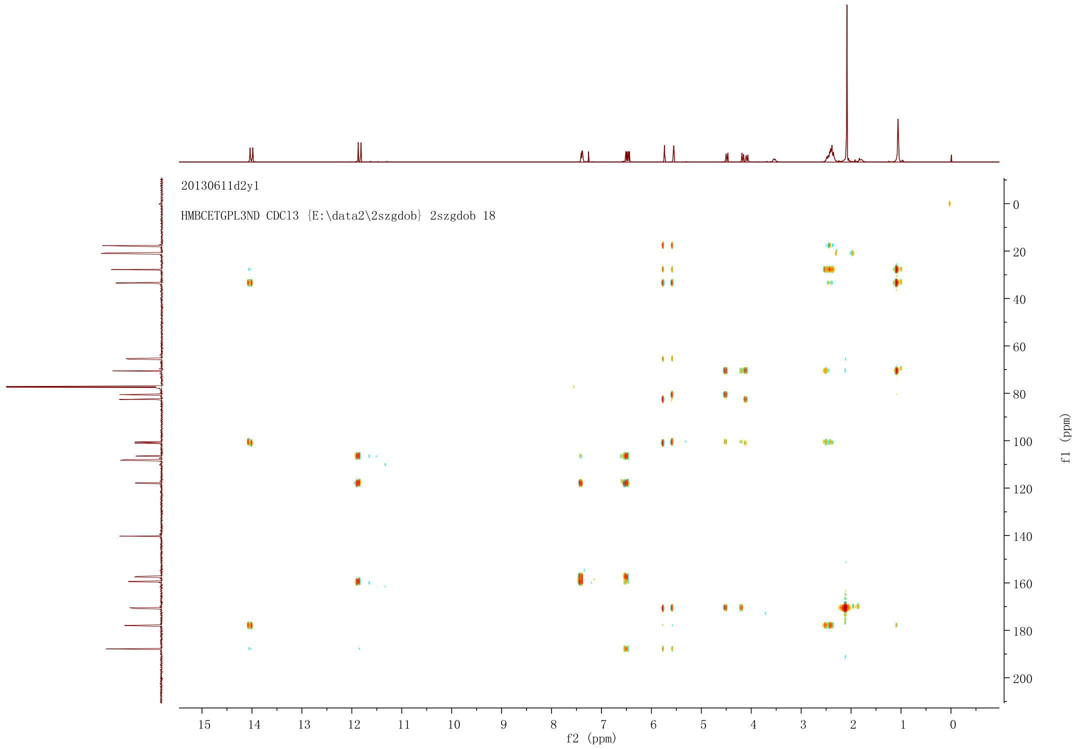

NOE

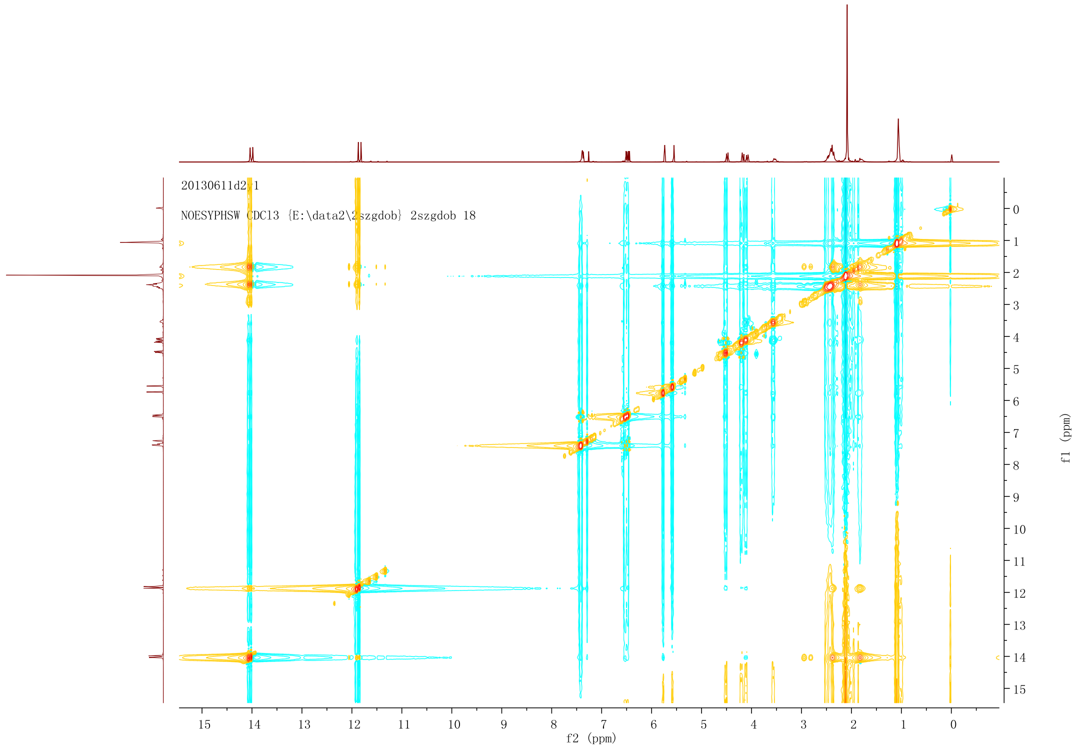

Figure S15. ^{1}H, ^{13}C, ^{1}H-^{1}H COSY, HSQC, HMBC, NOE of compound **6**.

^{1}H

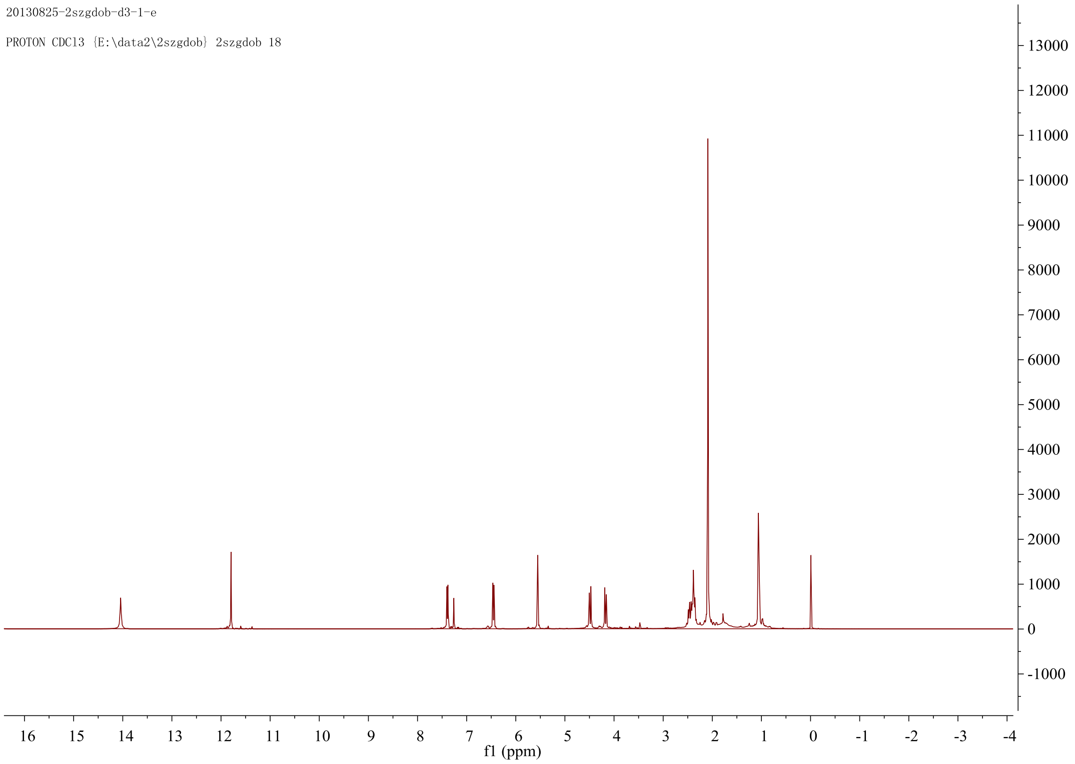

^{13}C

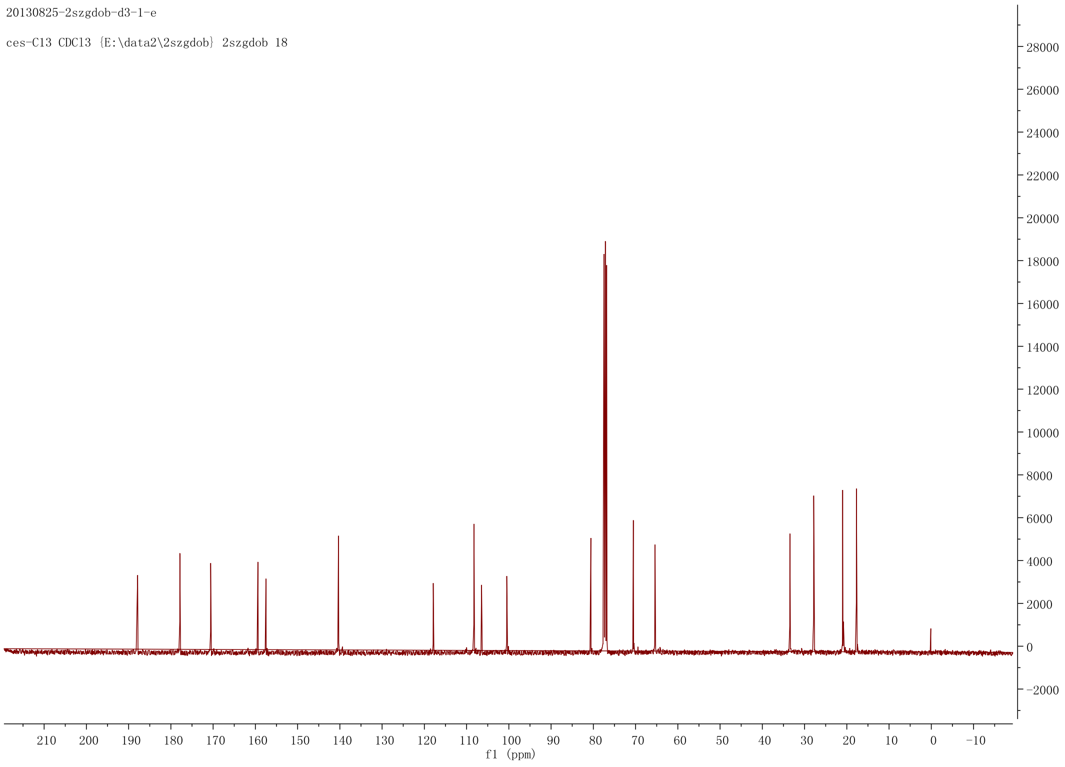

Figure S15. *Cont.*

^{1}H-^{1}H COSY

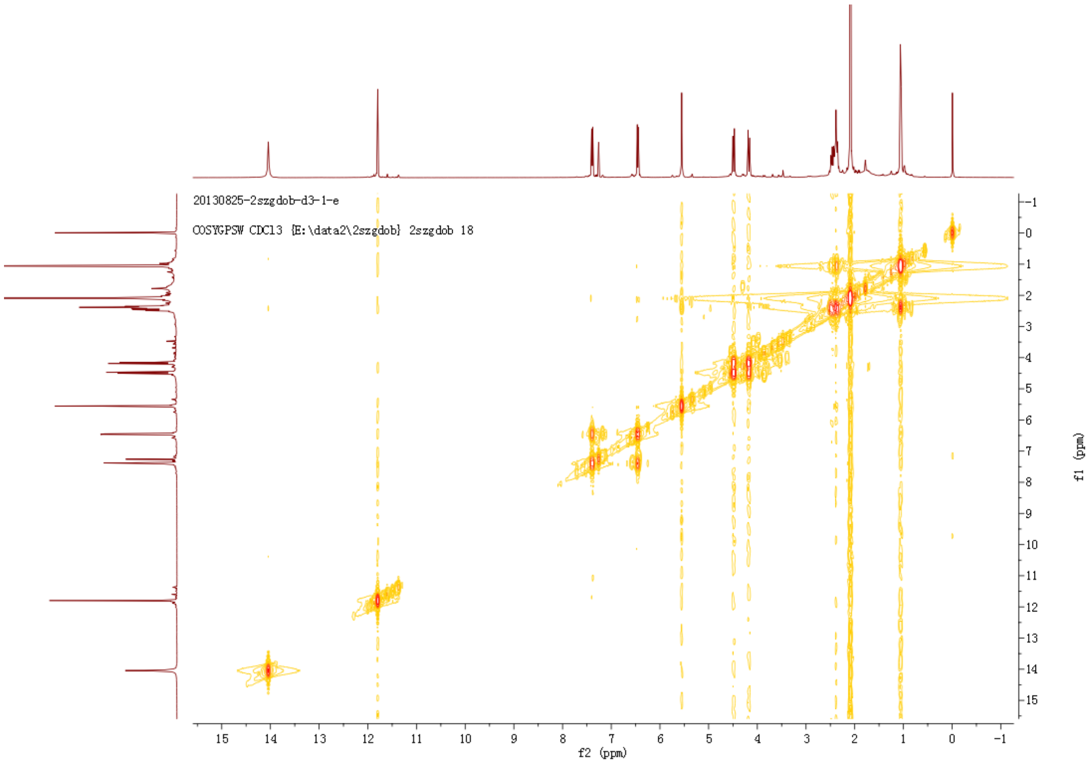

HSQC

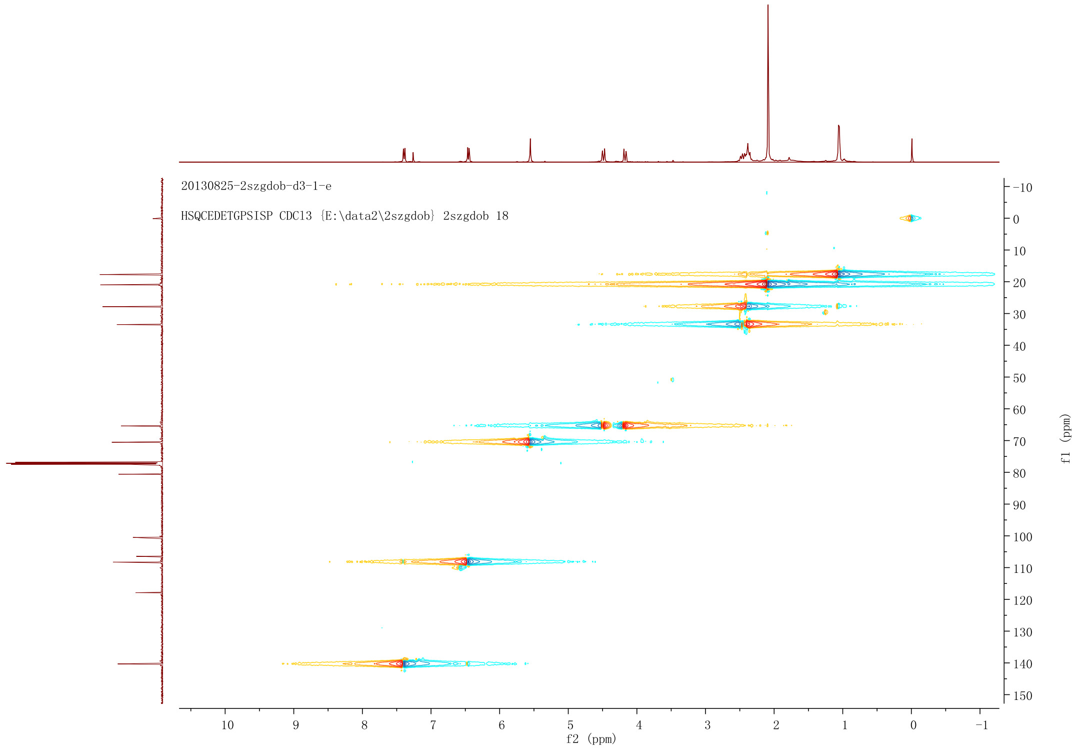

Figure S15. *Cont.*

HMBC

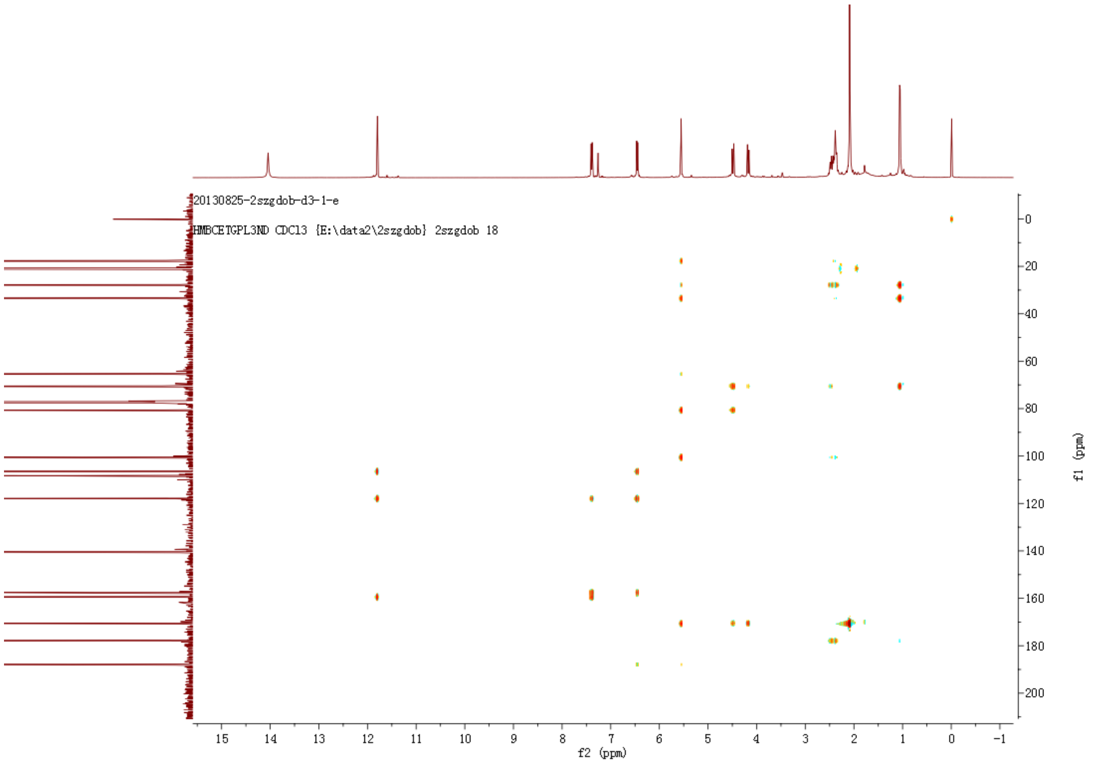

NOE

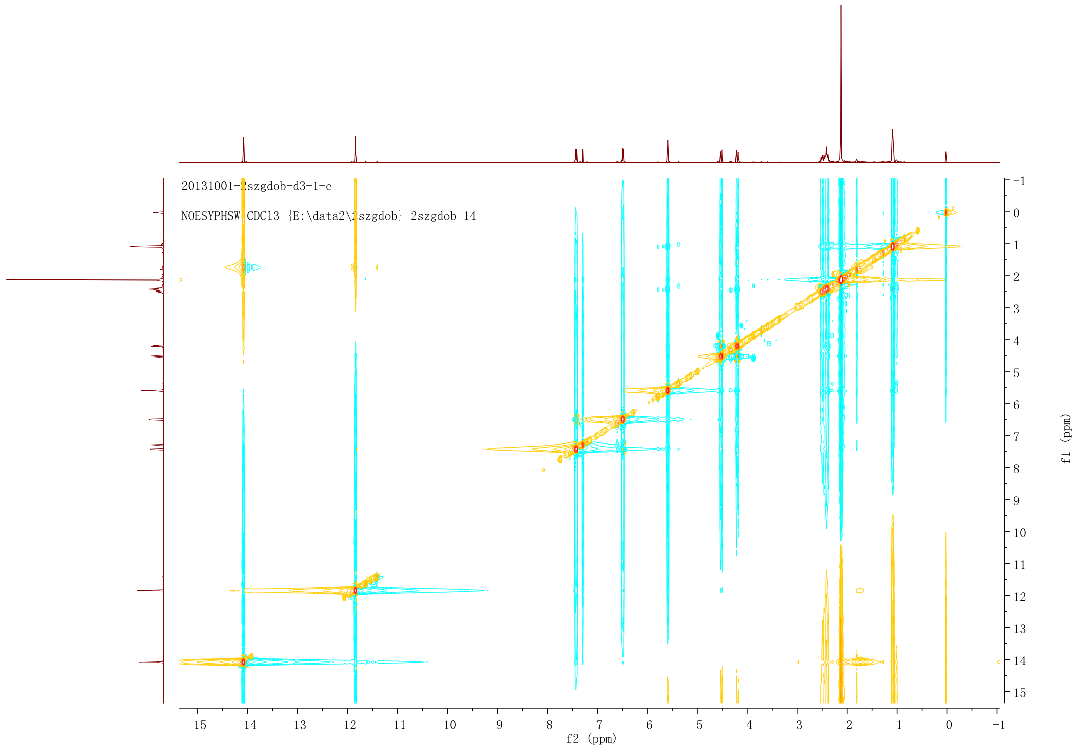

Figure S16. ^{1}H, ^{13}C, ^{1}H-^{1}H COSY, HSQC, HMBC, NOE of compound **7**.

^{1}H

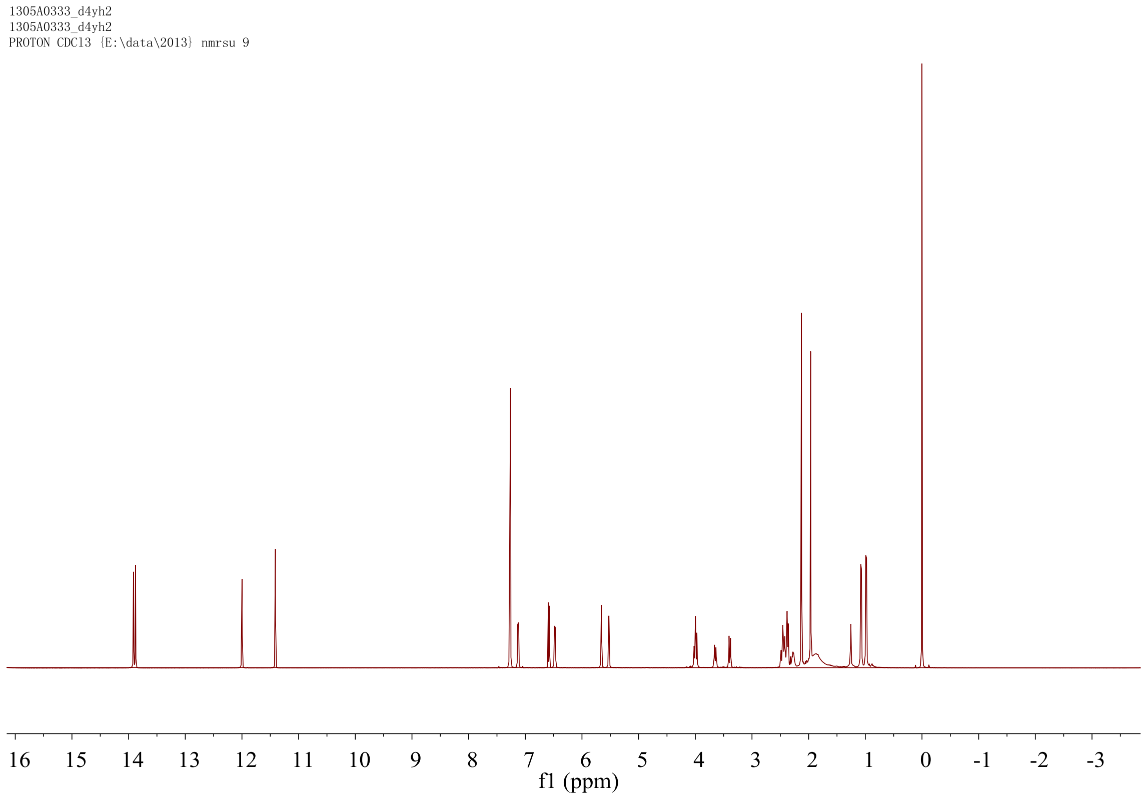

^{13}C

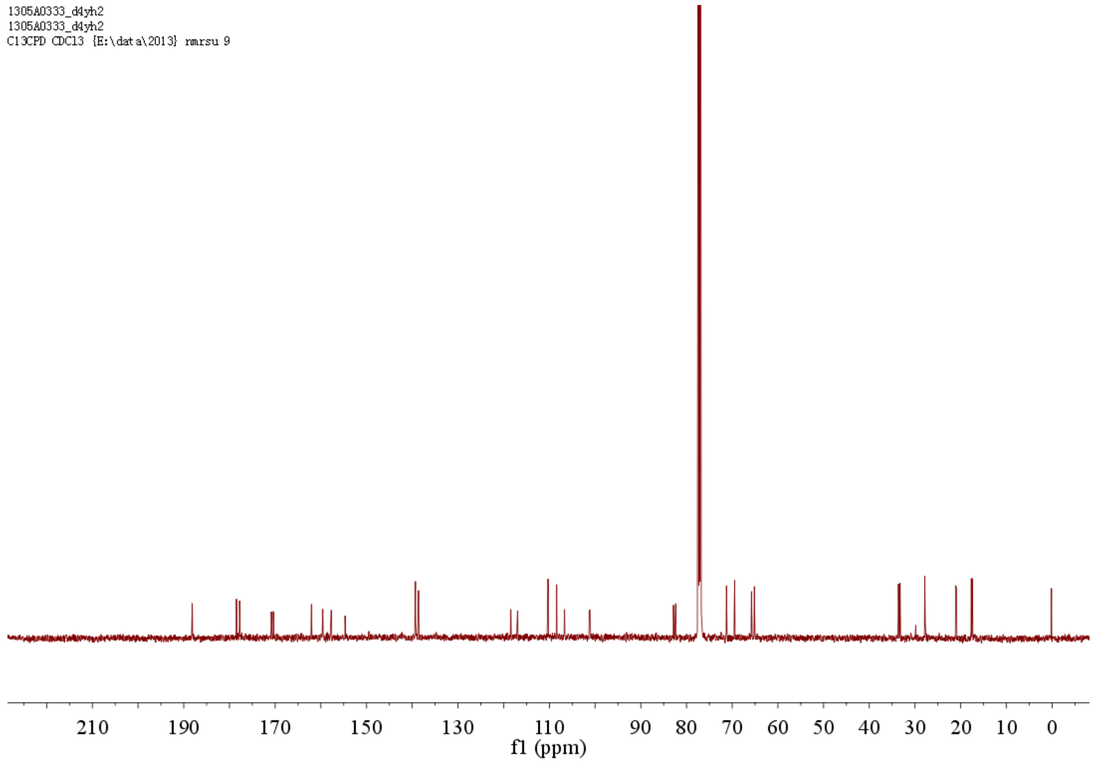

Figure S16. *Cont.*

^{1}H-^{1}H COSY

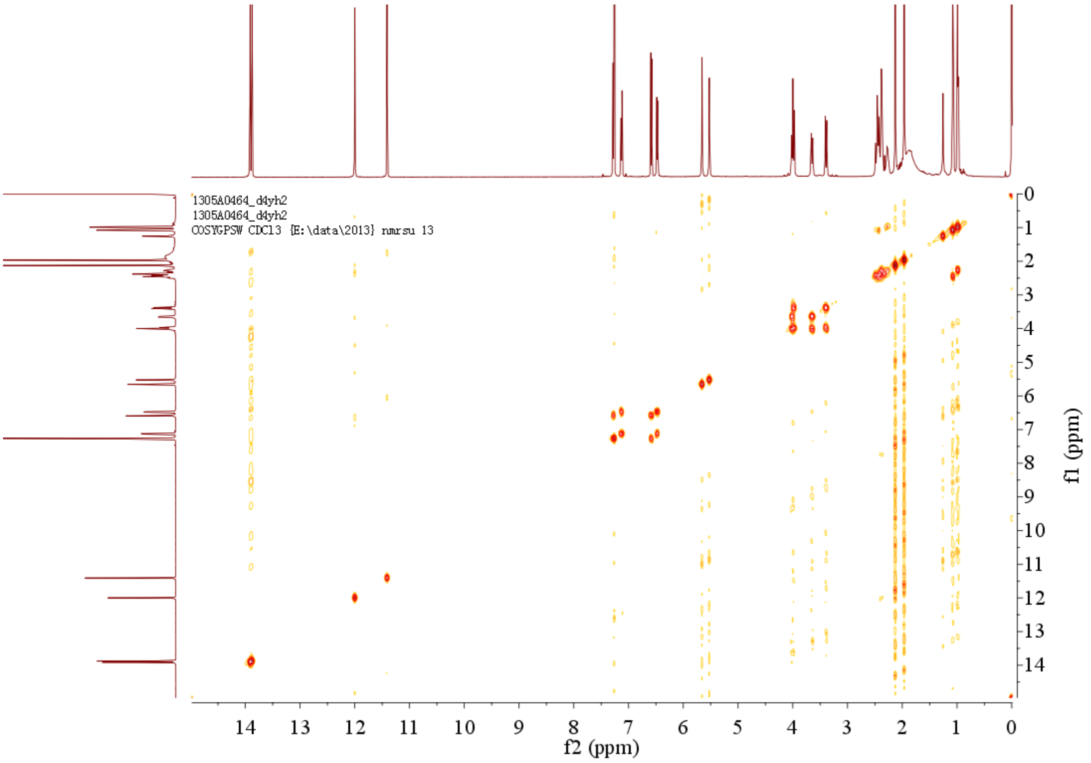

HSQC

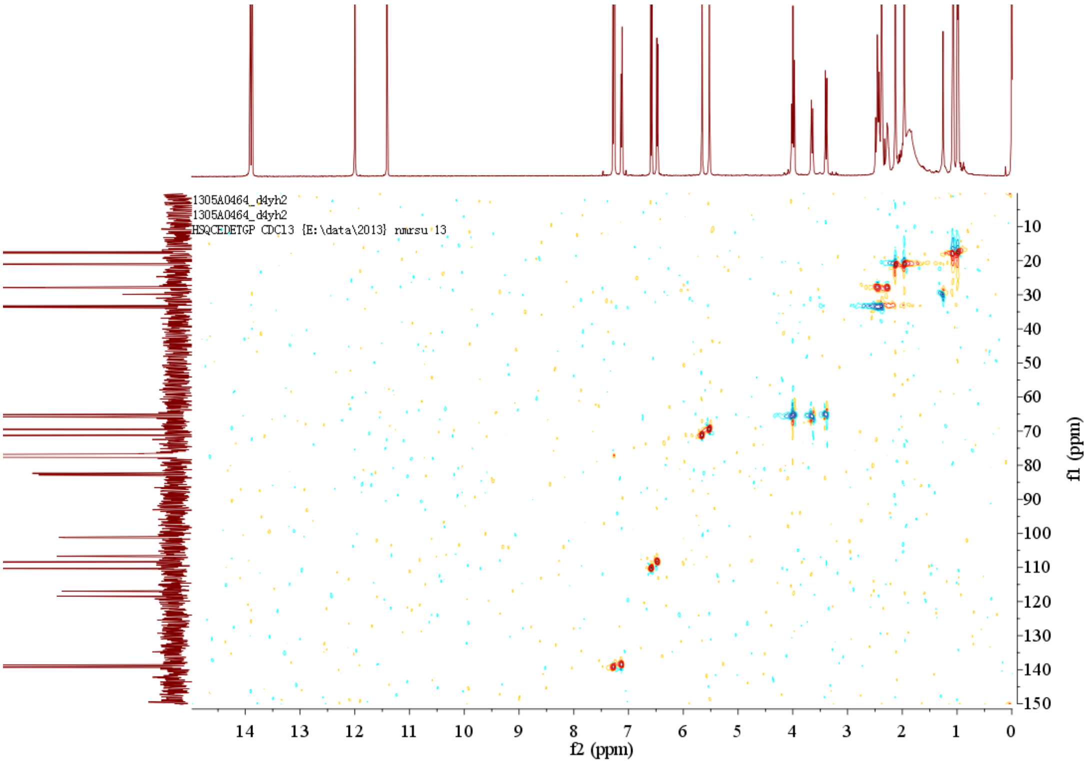

Figure S16. *Cont.*

HMBC

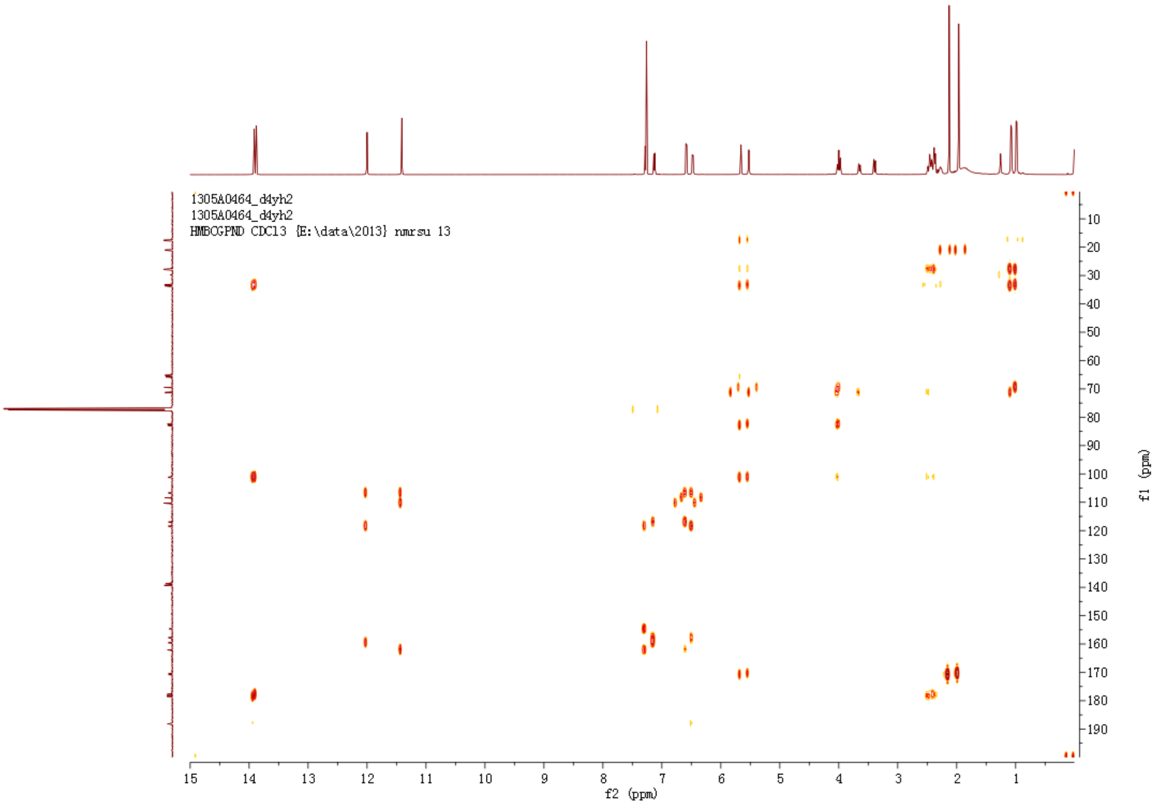

NOE

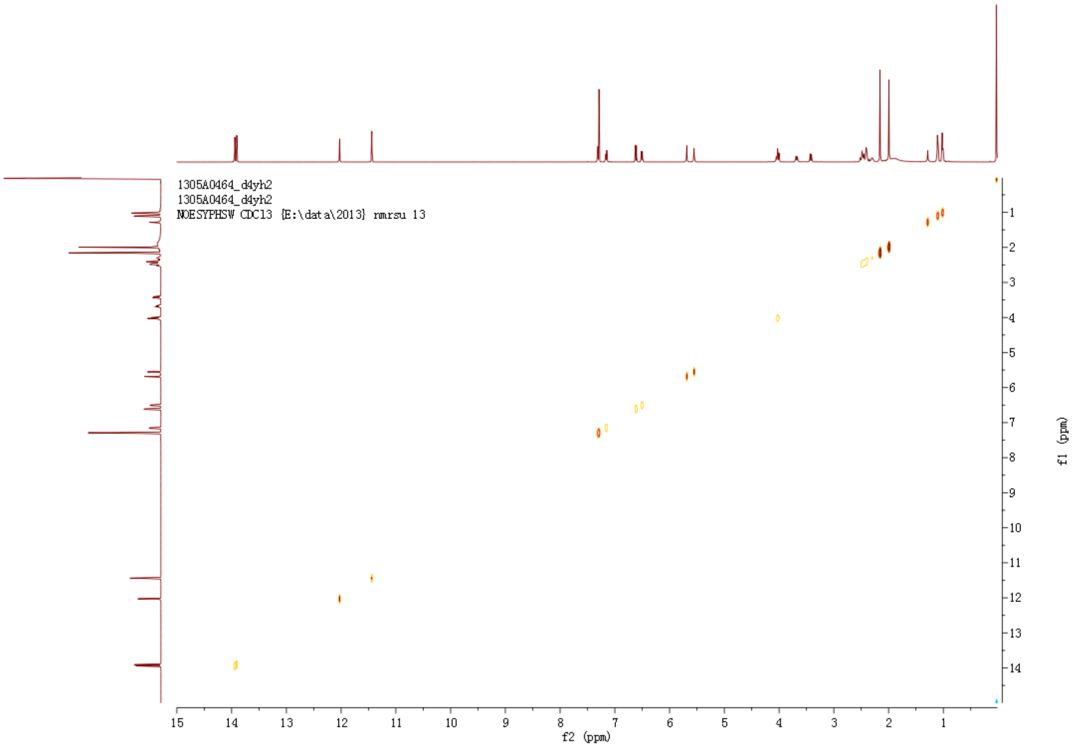

Figure S17. ^{1}H, ^{13}C, ^{1}H-^{1}H COSY, HSQC, HMBC, NOE of compound **8**.

^{1}H

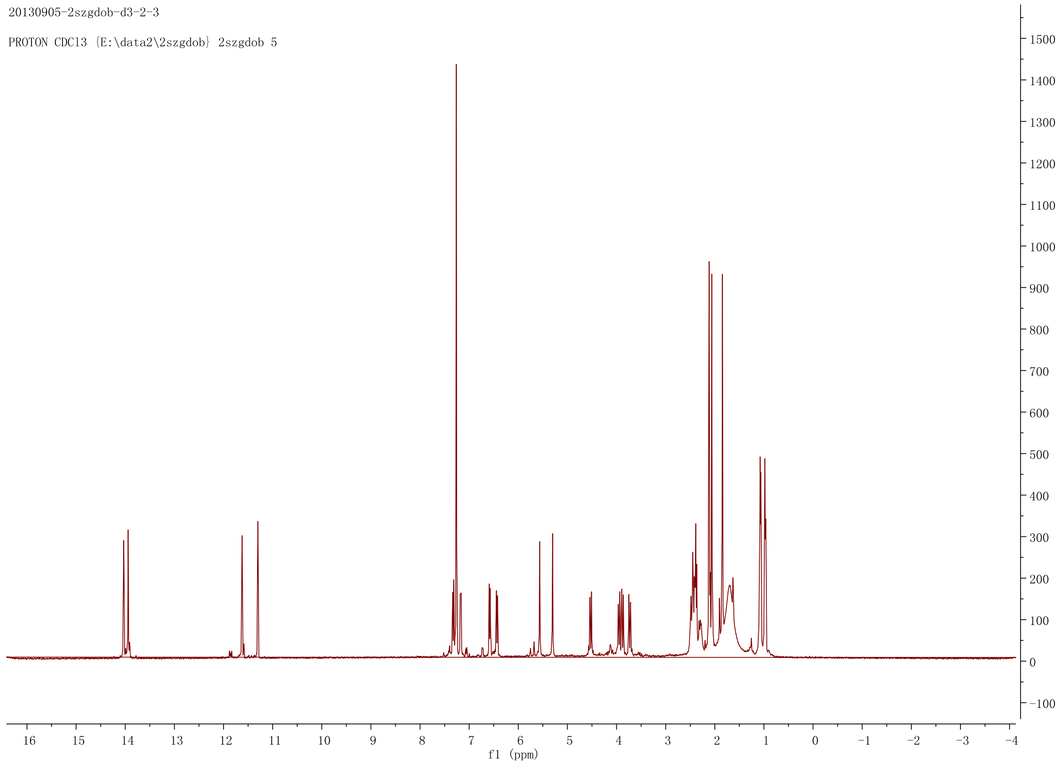

^{13}C

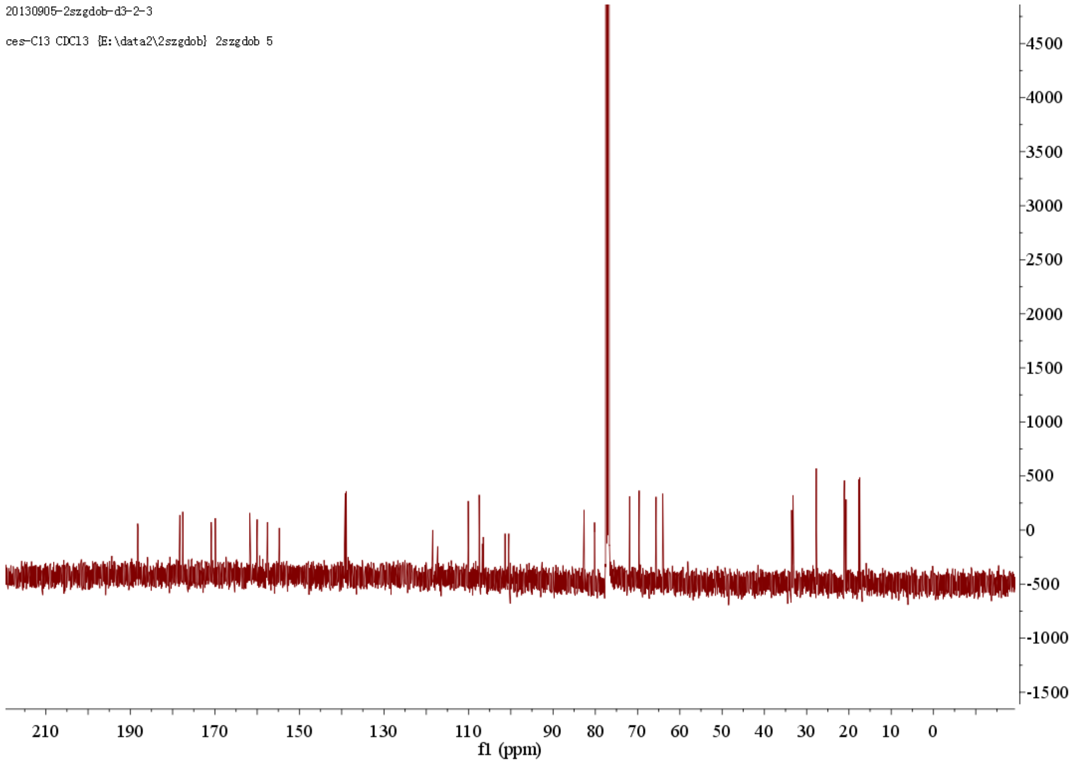

Figure S17. *Cont.*

^{1}H-^{1}H COSY

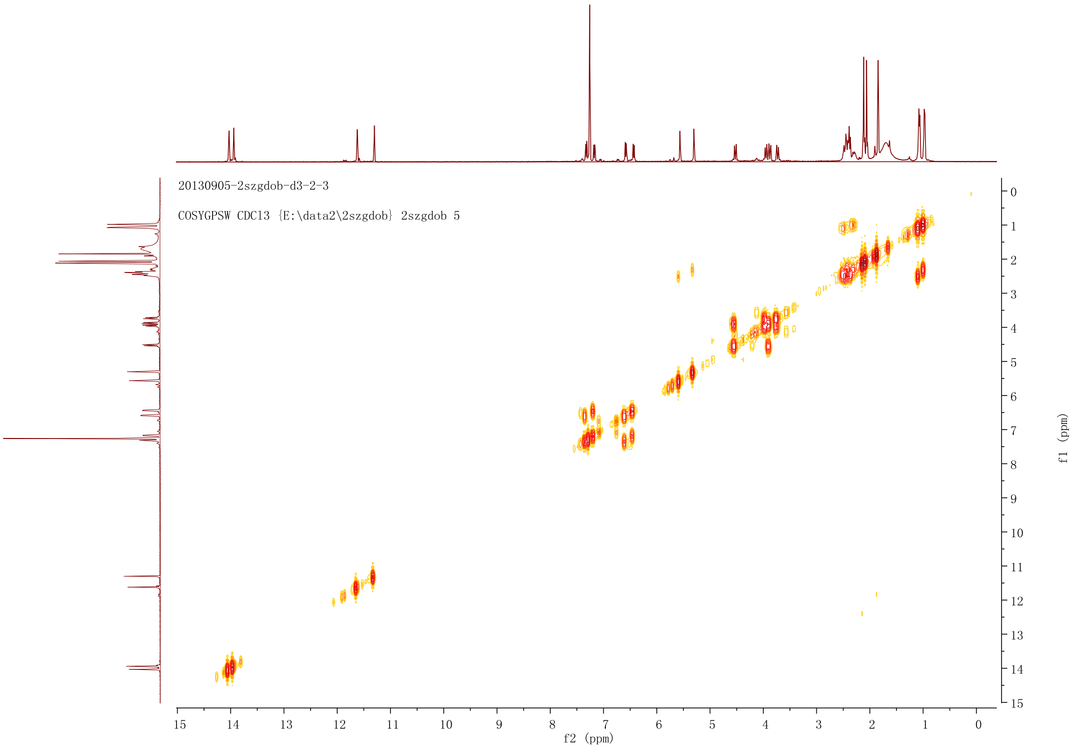

HSQC

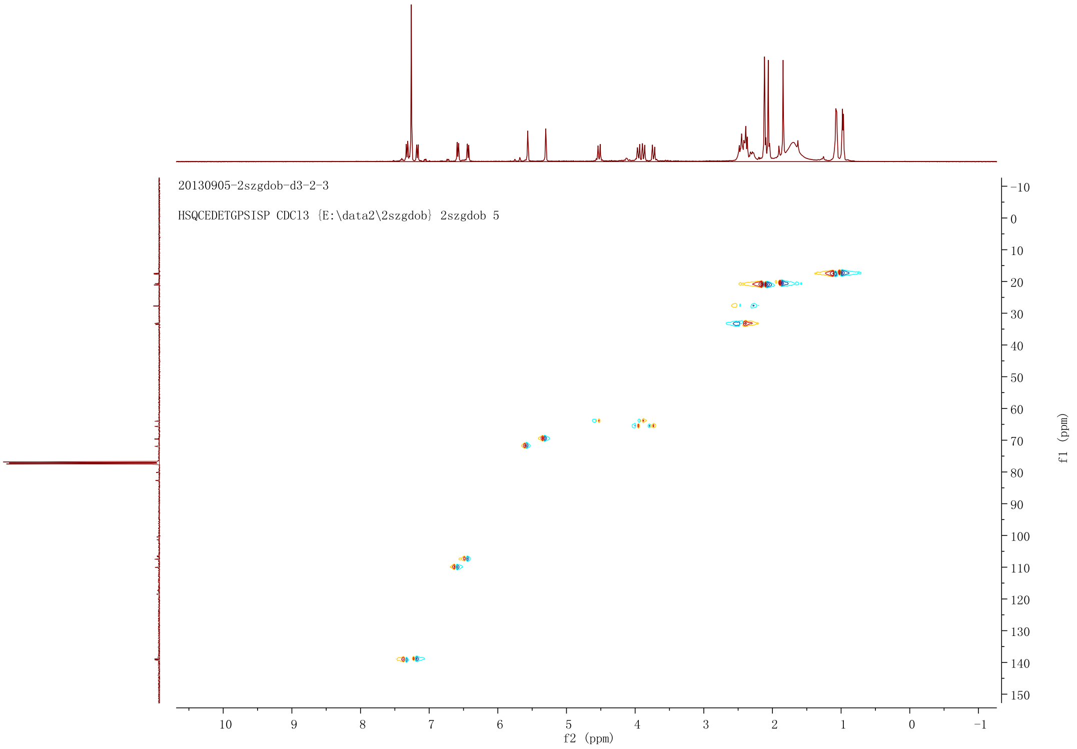

Figure S17. *Cont.*

HMBC

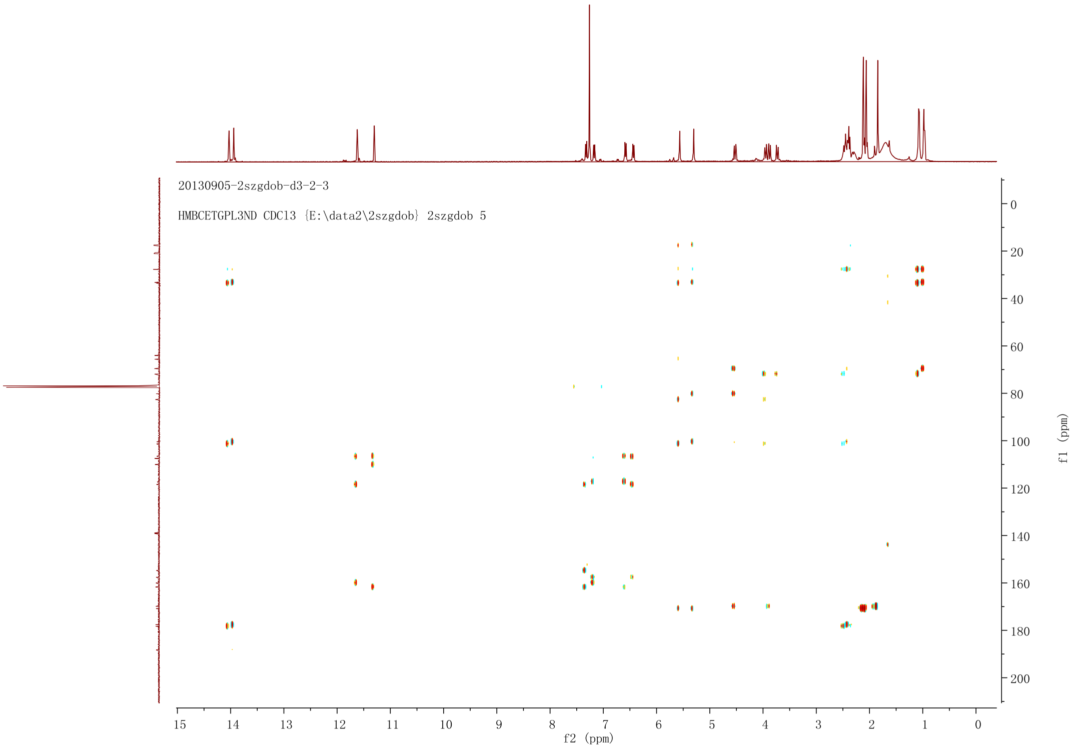

NOE

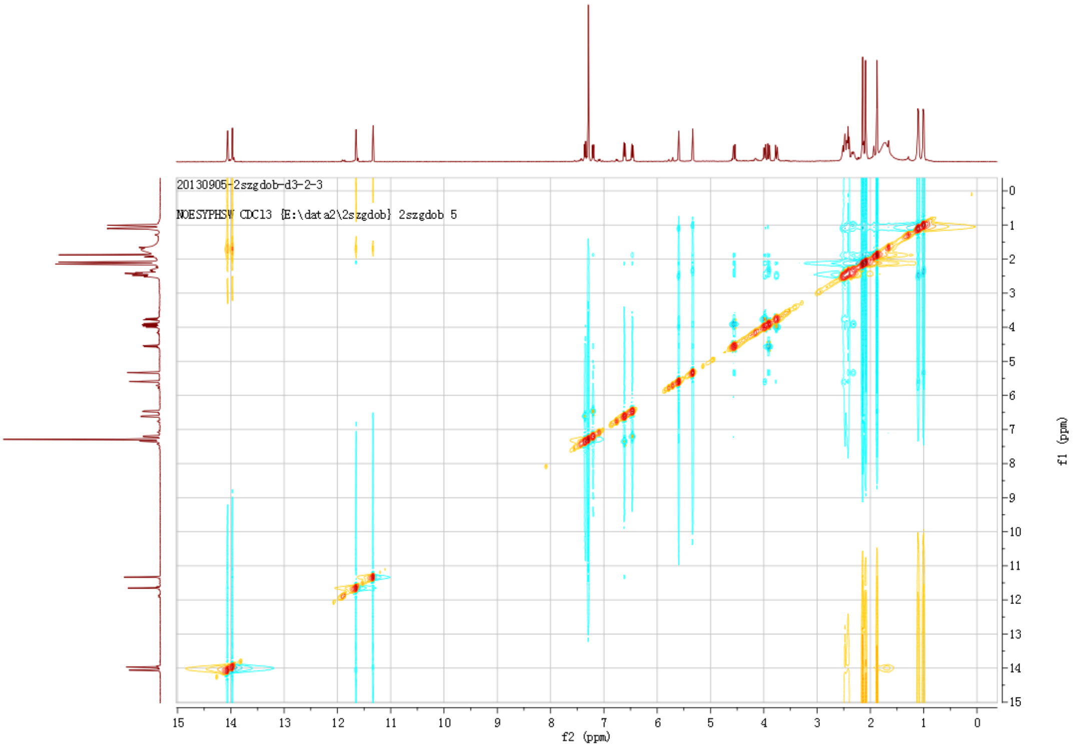

Supplement: Supplementary File 1 — Supplementary Information (PDF, 7459 KB) [file marinedrugs-11-04961-s001.pdf]
